# The development and maintenance of sex differences in dietary breadth and complexity in Bornean orangutans

**DOI:** 10.1007/s00265-021-03014-3

**Published:** 2021-04-21

**Authors:** Caroline Schuppli, S. Suci Utami Atmoko, Erin R. Vogel, Carel P. van Schaik, Maria A. van Noordwijk

**Affiliations:** 1 Max Planck Institute of Animal Behavior, Konstanz, Germany; 2 Leipzig Research Center for Early Child Development, University of Leipzig, Leipzig, Germany; 3 Department of Anthropology, University of Zürich, Zürich, Switzerland; 4 Fakultas Biologi, Universitas Nasional, Jakarta, Indonesia; 5 Department of Anthropology, Rutgers University, New Brunswick, NJ USA

**Keywords:** Diet repertoire, Feeding skill acquisition, Orangutan, Sex differences, Diet breadth, Diet complexity

## Abstract

**Abstract:**

Orangutans show a pronounced sexual dimorphism, with flanged males (i.e., males with fully grown secondary sexual characteristics) reaching twice the size of adult females. Furthermore, adult orangutans show sex-specific dispersal and activity patterns. This study investigates sex differences in adult foraging behavior and sheds light on how these differences develop in immatures. We analyzed 11 years of feeding data on ten adult female, seven flanged male, and 14 immature Bornean orangutans (*Pongo pygmaeus wurmbii*) at Tuanan in Central Kalimantan, Indonesia. We found that the diets of the adult females were significantly broader and required more processing steps before ingestion than the diets of flanged males. We also found evidence for a similar difference in overall diet repertoire sizes. For the immatures, we found that whereas females reached 100% of their mothers’ diet spectrum size by the age of weaning, males reached only around 80%. From the age of 4 years on (i.e., years before being weaned) females had significantly broader daily diets than males. We found no difference in daily or overall diet processing intensity of immature males and females but found preliminary evidence that immature males included fewer items of their mother’s diet in their own diets that were processing-intensive. Overall, our results suggest that by eating a broader variety and more complex to process food items, female orangutans go to greater lengths to achieve a balanced diet than males do. These behavioral differences are not just apparent in adult foraging behavior but also reflected in immature development from an early age on.

**Significance Statement:**

In many species, males and females have different nutritional needs and are thus expected to show sex-specific foraging behavior. Sex differences in several aspects of foraging behavior have been found in various species, but it remains largely unclear when and how those develop during ontogeny, which is especially relevant for long-lived altricial species that learn foraging skills over many years. In our study, we analyzed a cross-sectional and longitudinal data set containing more than 750,000 feeding events of adult and immature Bornean orangutans (*Pongo pygmaeus wurmbii*). We found that adult females had significantly broader and more complex diets than males. We also found that these differences started to develop during infancy, suggesting that immature orangutans prepare for their sex-specific foraging niches long before those become physiologically relevant while they are still in constant association with their mothers and before being frequently exposed to other role models.

**Supplementary Information:**

The online version contains supplementary material available at 10.1007/s00265-021-03014-3.

## Introduction

In many mammals, including primates, males and females lead fundamentally different lives. Males depend on physical strength to compete over access to females during a temporally limited prime time of their lives (van Noordwijk and van Schaik 2004). Females depend on being physically able to support pregnancy and lactation which may last over multiple years (Coelho 1974; Key and Ross 1999). These essentially different patterns of energy allocation have consequences for primate physiology and behavior on many levels. A male’s readiness to compete for and defend females requires large bodies, agility, and strength. Female reproduction, especially lactation is energetically costly (Lee 1987; Gittleman and Thompson 1988; Clutton-Brock et al. 1989; Lawler et al. 2005; van Noordwijk et al. 2013) and seems to require diets with specific nutrient composition during different stages of infant development (Bransford et al. 2019). There is ample evidence that these differing energetic and nutritional needs lead to different dietary requirements in adult males and females (Key and Ross 1999). Different dietary requirements have been found to be reflected in differences in foraging behavior (e.g., time spent feeding and feeding speed), diet composition , nutritional intake, and diet diversity (Clutton-Brock 1977; Hiraiwa-Hasegawa 1997; O'Mara and Hickey 2012, 2014; Vogel et al. 2017). These differences imply that males and females of the same species differ in the use of their foraging niche (Rose 1994; Melin et al. 2010).

In theory, sex differences in primate energetics should only become relevant during late juvenility (defined as the period between completed weaning and adulthood) or early adulthood because it is around this time that the two sexes begin to differ in size and energetics (Hiraiwa-Hasegawa 1997; van Noordwijk et al. 2002). However, over the last few decades, research has shown that immature individuals of many primate species acquire their foraging skills and dietary composition through an extended, often multi-year learning process during the developmental period and that diet learning is often heavily socially mediated (Watts 1985; Whitehead 1986; Galef and Giraldeau 2001; Matsuzawa et al. 2001; Biro et al. 2003; Tarnaud 2004; Ottoni et al. 2005; Rapaport and Brown 2008; Tarnaud and Yamagiwa 2008; Humle et al. 2009; Perry 2009; Coelho et al. 2015; Schuppli et al. 2016b). Immature males and females might therefore prepare for their sex-specific adult foraging niches before they reach juvenility or adulthood. However, to date, rather little is known about how differences in adult dietary requirements are reflected in primate development (but see Lonsdorf et al. 2004; Agostini and Visalberghi 2005; Gunst et al. 2008; Perry 2009; O’Mara and Hickey 2014; Lonsdorf 2017) and at what point sex differences in the use of the foraging niche emerge.

Sex differences in feeding behavior are expected to be most pronounced in species with a greater degree of sexual dimorphism and differences in activity patterns between the sexes. At the same time, the more a species relies on learning (as opposed to innate predispositions) to acquire their feeding skills, the more immatures should prepare for their sex-specific foraging niches before they actually become relevant (i.e., before immatures experience the direct energetic consequences of differing body size or behavior between the sexes). Orangutans (*Pongo* sp.) show one of the most pronounced sex differences in body size (Leigh and Shea 1995) and activity patterns (Mitra Setia et al. 2009; van Schaik et al. 2009; Vogel et al. 2017) of all primates. They also show extensive learning periods to acquire foraging skills (Schuppli et al. 2016a). This makes them a particularly appropriate taxon to examine the development of sex differences in feeding behavior.

Orangutan females are philopatric, with relatively stable home ranges (Ashbury et al. 2020), whereas males leave their natal area upon reaching sexual maturity to disperse into a new area (Nater et al. 2011; Arora et al. 2012; Nietlisbach et al. 2012). Adult males first go through a so-called “unflanged” phase (in most cases transient), during which they physically resemble females. After a period of continuing growth, they will transition into the flanged male phase with characteristic cheek flanges. Flanged males are around twice the size of adult females and have large home ranges with varying stability and, in most populations, actively compete over access to females (Atmoko et al. 2009; Mitra Setia et al. 2009; van Schaik et al. 2009; Spillmann et al. 2017). Overall, therefore, adult orangutan males of both morphs have significantly larger and less stable home ranges than females.

Orangutan diets are very broad and comprise many food items which require complex processing, i.e., food items often require multiple steps of manual and/or oral manipulation before they are ingested (van Schaik et al. 1996; Russon et al. 2009; Vogel et al. 2014; Schuppli and van Schaik 2019b). Although immatures reach diet repertoire sizes (i.e., the total number of different food items their diet) in the range of adult individuals at around age 7, when they are fully weaned (Schuppli et al. 2016a), they take 10–12 years to acquire adult-like intake rates (Schuppli et al. 2016a). Immature orangutans spend the first 8–10 years of their lives in permanent and close association with their mothers (van Noordwijk et al. 2009). During this time, socially mediated independent learning is the key element of dietary learning (Jaeggi et al. 2010; Schuppli et al. 2016b; Schuppli and van Schaik 2019a).

Another relevant aspect for behavioral adaptations of feeding behavior are the orangutans’ extreme energetic constraints. They are large-bodied, large-brained and arboreal, all of which impose relatively high energetic demands (Pontzer et al. 2010). At the same time, they live in forests which are often characterized by low and unpredictable fruit production (Marshall et al. 2009). As a likely consequence, orangutans have the slowest known mammalian reproductive rates, with multi-year lactation periods (van Noordwijk et al. 2013) and interbirth intervals of on average 7.6 years, (van Noordwijk et al. 2018). Along with their slow reproduction, energetically demanding lifestyle (e.g., a high degree of arboreality despite a large body size and long and intense periods of offspring care) and low-productivity habitat, orangutans have evolved physiological and physical adaptations to limit energy expenditure, use energy more efficiently and increase the range of food items they can eat (Taylor et al. 2008; Narita et al. 2010; Vogel et al. 2014; Pontzer et al. 2016; Vogel et al. 2017). In addition, orangutans may also have evolved behavioral adaptations that allow them to flexibly adjust foraging behavior to changing individual needs throughout life. First, due to their differing energetic and nutritional needs, males and females might differ in the strategies they use to construct their best possible diets. Previous studies showed that adult males and females differ in aspects of energy intake and the nutritional composition of their diets, with flanged males having lower energy intake and less fat and protein in their diets than adult females when controlling for metabolic body mass (Knott 1998; Vogel et al. 2017). Second, adult orangutans might use sex-specific strategies to cope with fluctuations in food availability in that flanged males reduce their energy intake less than females during periods of low food availability (Knott 1998; Harrison et al. 2010).

In terms of the sex specific use of their foraging niche, following the concepts of energy maximization and time minimization (Schoener 1971; Hixon 1982), female and male adult orangutans may pursue different strategies. To be able to support pregnancy and lactation under the experienced energetic contstaints, female orangutans are likely to be energy maximizers, i.e., they are expected to aim at maximizing food-energy input and diet quality. Both flanged and unflanged orangutan males follow time intense socio sexual strategies. Flanged males attract females and then engage in mate-guarding associations and unfIanged males actively search for and pursue females (Delgado Jr and van Schaik 2000; Atmoko and van Hooff 2004). Therefore, contrary to adult females, male orangutans may aim at limiting the time they spend feeding to be able to spend more time monitoring and pursuing females (van Schaik et al. 2009). This could mean that adult female and male ourangutans might prefer food items which differ in terms of their processing time and nutritional value.

In this study, we investigated sex-specific feeding behavior in wild Bornean orangutans (*Pongo pygmaeus wurmbii*) and its development during the immature period. As a first step, we compared the diets of adult females and flanged males. We expected that adult female orangutans, because they have to support pregnancy and lactation, depend on specific nutrient composition and thus need broader diets. In contrast, because of their large body size flanged males have overall higher energetic maintenance requirements and spend more energy during activity. They should therefore focus on rapidly harvested, if nutritionally less rewarding, food items, which will bias their chocies toward a narrower diet, containing items with less processing time. Such a foraging strategy allows them to reduce the high energy expenditure linked to travel (Vogel et al. 2017). Overall, we therefore predict that females will go to greater lengths to achieve broad diets (i.e., diets that are comprised of a larger number of different food items) and that males are more likely to pass up food items that require time-intense processing and thus most likely have low energy return rates in relation to their processing time (henceforward called “complex items”). Based on the fact that immature orangutans learn the composition of their diets and how to process the food items in their diets over a lenghty learning period, we also predict that the sex differences in dietary requirements will already be manifested in the feeding behavior of young immatures.

Specifically, we predict that among adults, (1) females will show broader daily diets (i.e., they will eat more different food items per day) than males, whereas (2) flanged males will tend to consume less complex food items and thus show less complex daily diets. For the immatures we predict that (3) female immatures will develop broader and (4) more complex diets than their male peers.

## Methods

The data for this study were collected at the Tuanan research station in Central Kalimantan, Indonesia from 2003 to 2018 during full-day focal follows (i.e., data on the behavior of one focal individual was collected) of adult females, flanged males, and immatures. During these follows, the activity of the focal animal was noted through observation scans at two-minute intervals. It was not possible to record data blind because our study involved focal animals in the field. For all food items, the species and the part eaten (i.e., leaves, fruits, bark, pith or other vegetative plant material and insects or insect products) were noted. We refer to a specific food species – part combination as a “food item”. We included data from all trained observers that had collected data at the site (Table ESM 1 lists all food items that occurred in the data of this study).

The processing complexity of the food item was assigned according to the number of steps the orangutans use to process this food item before swallowing (e.g., biting open, peeling, spitting seeds out; see Table ESM 2 and Schuppli et al. 2016a for details) and ranged from 0 to 4. The frequency distribution of the processing steps in the data used in this study is summarized in Table ESM 3.

### Data set on the adult focal animals

Because adult females have higher site fidelity, our data contain more follow hours on individual adult females compared to individual adult males. Our data also contain more follow hours on resident flanged males, defined as flanged males that have been seen in the area for at least six consecutive years, than on other flanged males and unflanged males, so we chose to analyze only data on resident flanged males. By only including resident flanged males, we not only obtained a high data density for each individual but also maximize comparability of the two sexes in terms of knowledge of the local area.

To compare daily diet processing complexity (i.e., the average number of pre-ingetive processing steps of the food items eaten in a day, weighted by the time that was spent feeding on these items) and daily diet breadth (i.e., the average number of different food items eaten in a day) of flanged males and adult females, we used feeding data collected during nest to nest, full day follows on 10 adult females and seven flanged males. A total of 30,786 follow hours, collected during a total of 2787 follow days (ranging from 25 to 519 follow days per individual, mean = 164) and containing a total of 499,514 feeding bouts (i.e., 2-min scans at which the individual was feeding, ranging from 4137 to 100,345 total feeding bouts per individual, mean = 29,383) were used.

To compare overall diet repertoire sizes of flanged males and adult females, we counted the total number of food items in their recorded feeding bouts. The different food items vary in their frequency in the population’s diet (i.e., how many feeding bouts were on this item). A total of 318 different food items were recorded in the feeding data of the adult focal individuals of this study and their frequencies range from 0.0002 to 12% (based on 499,720 feeding bouts, see Fig. ESM 1 and Table ESM 1). Furthermore, the availability of the food items in the habitat varies within and between years. Consequently, diet repertoire sizes of the individuals are highly dependent of the follow effort (i.e, the number of hours the individual was followed). Therefore, we investigated cumulative diet repertoire sizes over increasing follow effort. We used the Michaelis-Menten saturation curve, which is commonly used to model species accumulation curves, to model the effect of the follow effort on diet repertoire size (Keating and Quinn 1998; Lopez et al. 2000). We maximized the data density of our data set by using all data available on each of our adult focal animals, including feeding data collected during partial follows (where the focal animal was only found during the day and thus the follow started later, or the focal animal was lost during the day which meant the follow stopped earlier). To be able to do this comparison reliably, we only included individuals for whom we had data collected in at least six consecutive years. This resulted in a total of 33,591 follow hours (ranging from 625 to 6595 hours per individual, mean = 2584) of six females and seven flanged males.

### Data set on the immature focal animals

To investigate the acquisition of the diet repertoires during development (i.e., when diet repertoires are learned) we examined the diets of the immatures at different stages throughout their development. At Tuanan, after weaning, which is on average at 7 years (van Noordwijk et al. 2018), immatures remain in constant association with their mothers for 1–3 years. Throughout the manuscript, we refer to individuals from birth until the end of the constant association with their mothers as dependent immatures. During the dependency period, we have large amounts of simultaneous data on the immature and its mother available whereas after the end of the constant association, these become sparser, especially for the male immatures who soon after leave their natal area. Consequently, most of our analyses on the immatures focus on the dependency period.

For the development of the overall diet repertoire size, we compared the number of food items a mother and her offspring would eat within each age year of the offspring (i.e., 0–1 years, 1–2 years, etc.). Immatures were followed simultaneously with their mothers by a team of 2–4 observers for maximally 10 days within a month. Food item counts are heavily dependent on follow effort and current food availability (which was assessed via the number and variability of fruit bearing trees and liana present in the study area). Therefore, to assess the diet repertoires of the immatures we always used the diet repertoire of the mother, collected in the same time period, as a reference. We later used the mothers’ food item count as an offset in our statistical model (see below). For each age year of the immature, we computed the cumulative total number of food items eaten by the immature and its mother. For this analysis of the development of overall diet repertoire size, we had data on 14 immatures (7 females, 7 males, aged from 0 to 12 years) and their 8 mothers available. For each immature we had data from 3 to 11 (mean = 6.929) different age years available, resulting in a total of 97 data points (i.e., yearly overall repertoire sizes).

To investigate how daily diet breadth and daily diet processing complexity develop, we used feeding data collected during nest to nest, full day follows on our dependent immature focal animals. A total of 23,573 follow hours, collected during a total of 2094 follow days (ranging from 10 to 498 follow days per individual; mean = 161) and containing a total of 284,829 feeding bouts (ranging from 457 to 77,130 total feeding bouts per individual, mean = 21,910) were used. To compare the overall diet processing complexity and diet breadth of mothers with their female and male offspring, we used all feeding data available for each mother during the time spans she was with the respective dependent offspring. To avoid confounding effects of the offspring age, we only included the 11 mother offspring pairs for which we had data on the whole dependency period of the offspring available and additionally included the age at which the dependency period ended.

### Statistical analyses

We conducted all statistical analyses and plots in the R programming language (R Development Core Team 2019). To assess the effect of the predictors sex and age on our response variables we used generalized linear mixed models (GLMM) with a Gaussian (in the case of the continuous data) or Poisson (in the case of count data) family distribution, as implemented in the package *lme4* (Bates et al. 2015), using maximum likelihood estimators. In all analyses, the individual was included as a random factor, to account for the fact that individuals occurred multiple times in the data set. Likewise, to account for possible confounding autocorrelation effects of data that were collected closely in time, the combination of year and month that the data were collected in was included as a random effect for all analyses in which the data were based on daily values. Age and follow effort (which was a control variable) were included as a linear factor, sigmoid factor or as a function of the Michaelis-Menten equation (Lopez et al. 2000), depending on the nature of the plotted raw data. For the Michaelis-Menten equation (which was used for the effect of increasing follow effort on cumulative overall diet repertoire size of adult females and flanged males) we assessed the fit in a first step via a non-linear least square regression model and the drc package (Ritz et al., 2016) before we included the effect in the final GLMM. We followed a full model approach whereby we first always compared the full model to the null model (which comprised only the control predictors and random effects) with a likelihood ratio test (LRT) via the *anova* function (Fox 2015; Dobson and Barnett 2018). In case the full model was supported, we assessed the significance of the effects via their p-values in the full model (in the case of the GLMM with a Gaussian family distribution the p-values were computed with the *cf*-test function; (Hothorn et al. 2021).

All model fits were examined visually to assess whether they satisfied model assumptions (for the models with a Gaussian family distribution normally distributed model residuals, homogeneity of the variance, and normally distributed random effects) and to check for the presence of influential observations (Harrell Jr 2015). The overall fit of the model was assessed via conditional pseudo delta R^2^, retrieved via the MuMlm package (Bartoń 2009; Nakagawa et al. 2017). We assessed the stability of all our mixed models on the level of the random effects by excluding individuals and data collection month-year combinations one at a time. We found that the direction of the random effects was consistent in all the supported mixed models. For all models with a Poisson family distribution, we tested for overdispersion and report the dispersion parameter following Mundry (2014).

## Results

### Diet breadth and complexity of adults

**Fig. 1:**
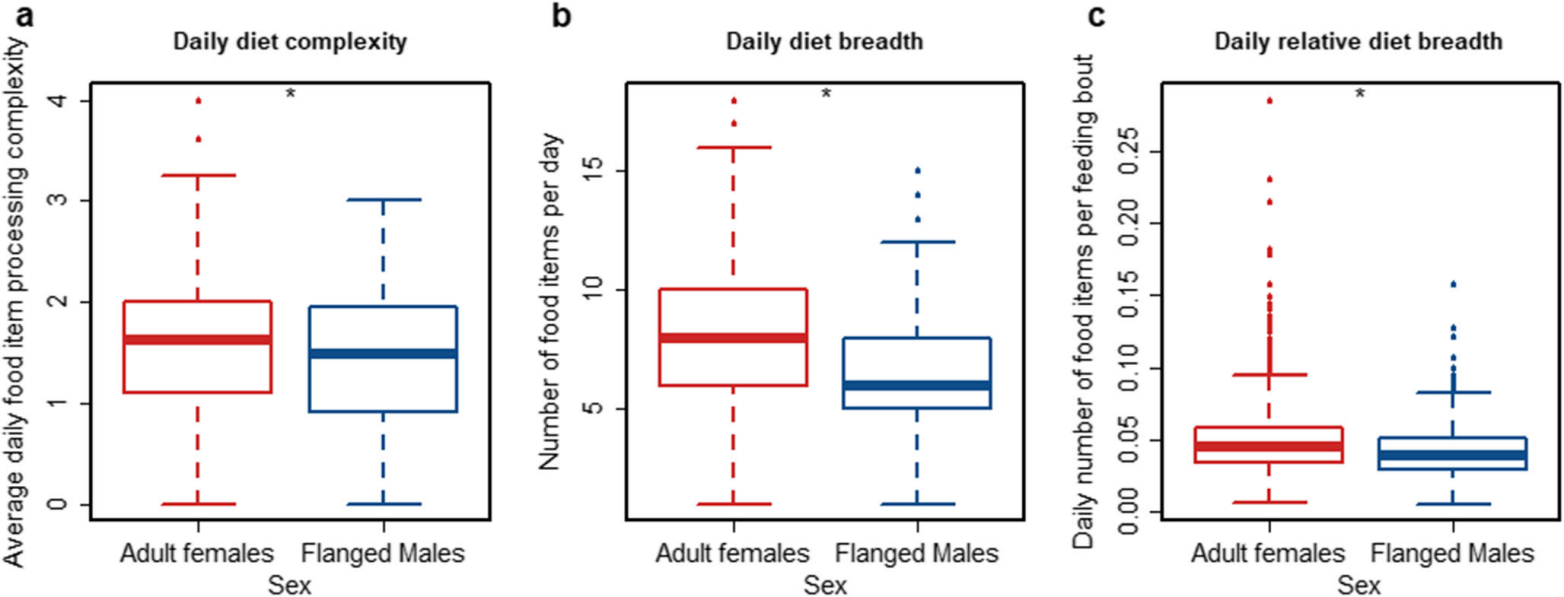
Diet processing complexity and daily diet breath of adult females and flanged males: average daily food item processing complexity (**a**), daily number of different food items (**b**) and relative daily number of food items (number of food items divided by number of feeding bouts on that day, (**c**). The asterisks indicate significant differences on the five percent level. The boxes indicate the inter quartile range (IQR), the central line depicts the median, the whiskers extend to 1.5*IQR and outliers are plotted individually

We found that the full models (which included the predictor sex, see Table 1a–c for model details) fitted the data better than the null models (which only included the random effects and control predictors) for average daily diet processing (LRT full model versus null model: Chi-square = 6.097, *P* = 0.014), average daily diet breadth (LRT full model versus null model: Chi-square = 5.734, *P* = 0.017) and relative daily diet breadth (corrected for the number of feeding bouts; LRT full model versus null model: Chi-square = 4.021, *P* = 0.045). The full models revealed that average daily diet processing complexity was significantly higher for adult females than for flanged males (Fig. 1a, Table 1a) that adult females had significantly broader daily diets (i.e., they ate a larger number of different food items per day) than flanged males (Fig. 1b, Table 1b), and that adult females showed a significantly higher relative daily diet breadth than flanged males (i.e., they ate a larger number of different food items per day controlled for the number of feeding bouts; Fig. 1c, Table 1c).

**Table 1:** Sex differences in the daily diet processing complexity and breadth of adult females and flanged males: The effects of sex on average daily food item processing complexity (a), daily number of different food items eaten (b) and the relative daily number of different food items (number of different food items controlled for the number of feeding bouts (# F bouts) on that day c), analyzed with GLMMs. R^2^ refers to conditional pseudo delta R^2^. Significant *P*–values of predictors are indicated with bold font

Nr.	Response variable	Factor	Factor type	Estimate	Std-Error	P	R^2^	Dispersion parameter	Family distribution
a)	Average daily food item processing complexity	Intercept	Intercept	1.702	0.025	<0.001	0.131		Gaussian
		Sex (m)	Predictor	−0.200	0.081	**0.013**			
		Year month	Random	-	-	-			
		Individual	Random	-	-	-			
b)	Daily number of different food items	Intercept	Intercept	2.077	0.031	<0.001	0.214	0.766	Poisson
		Sex (m)	Predictor	−0.122	0.047	**0.010**			
		Year month	Random	-	-	-			
		Individual	Random	-	-	-			
c)	Daily number of different food items	Intercept	Intercept	-3.076	0.034	**<0.001**	0.296	0.963	Poisson
		log(# F bouts)	Control (Offset)	-	-	**-**			
		Sex (m)	Predictor	-0.104	0.049	**0.0345**			
		Year month	Random	-	-	-			
		Individual	Random	-	-	-

Similar to previous studies (van Schaik et al. 2009; Vogel et al. 2017), we found no evidence for differences in overall time spent feeding per day between flanged males and adult females, as the full model (GLMM_Poisson_: daily feeding bouts ~sex + (1|individual) + (1|year month)) did not fit the data better than the null model (which only included the random effects; LRT full model versus null model: Chi-square = 0.459, *P* = 0.498, Fig. ESM 2a). However, for length of the active period, the full model (which included the predictor sex, see Table ESM 4 for model details) fitted the data better than the null model (which only included the random effects; LRT full model versus null model: Chi-square = 6.130, *P* = 0.013). The full model revealed that females had longer daily active periods than males (Fig. ESM 2b, Table ESM 4). For the relative time of the active period spent feeding we found that the full model (GLMM_Poisson_: daily feeding bouts ~sex + offset (log( daily active period)) + (1|individual) + (1|year month)) did not fit the data better than the null model (which only included the random effects and control predictor, i.e., offset; LRT full model versus null model: Chi-square = 0.215, *P* = 0.643, Fig. ESM 2c).

**Fig. 2:**
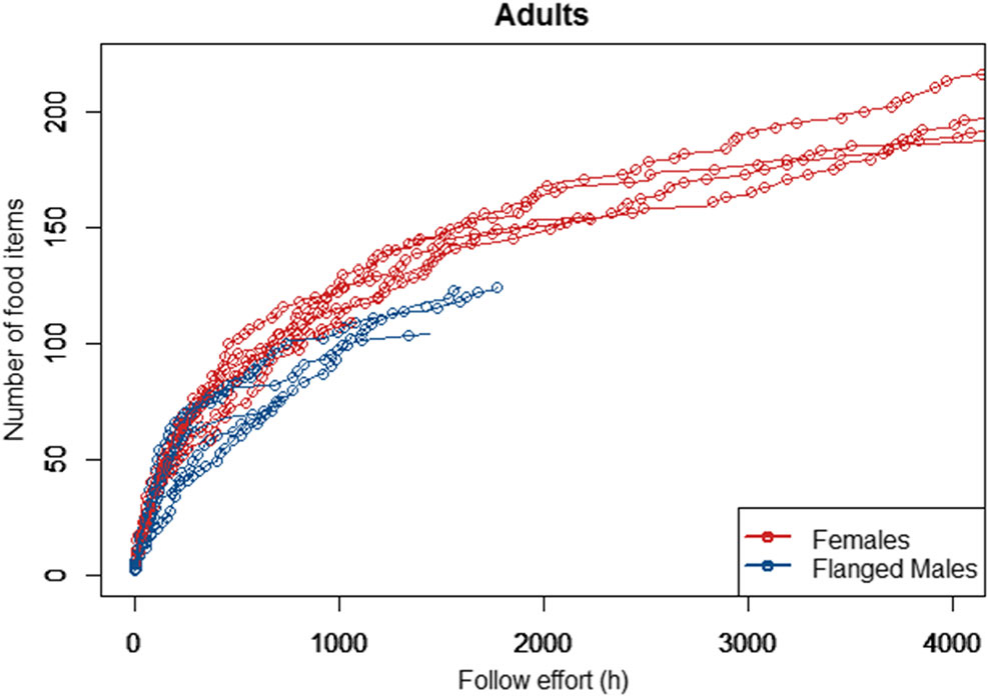
Adult overall diet repertoires: Diet repertoire size as a function of follow effort for adult females and flanged males

**Fig. 3:**
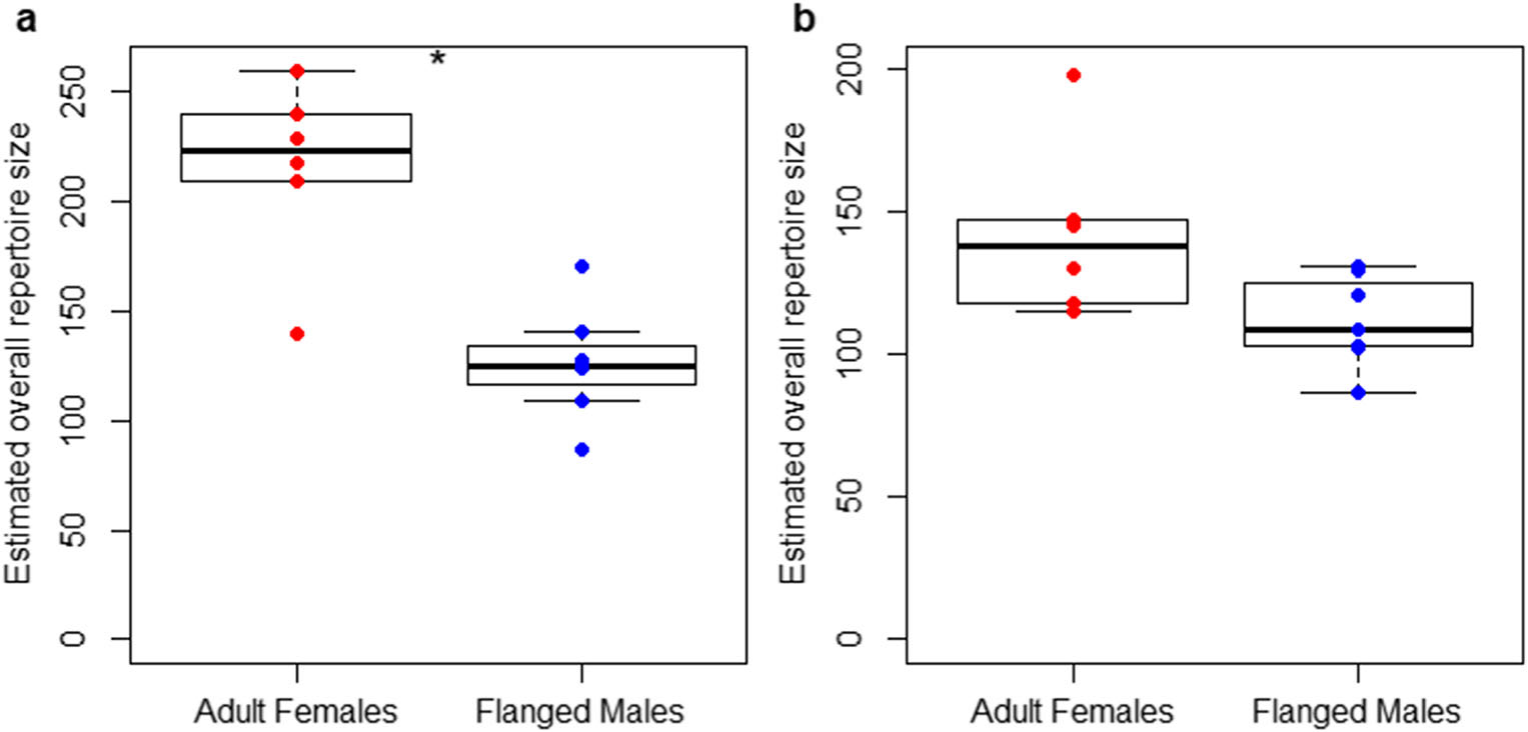
Adult estimated total diet repertoires: Estimated sizes of the total diet repertoires of adult female and flanged males, estimated via a function of the Michaelis-Menten equation based on (**a**) the complete data sets available of each individual and (**b**) the first 625 follow hours available of each individual (which is the number of total follow hours available on the individual that was followed the least). The asterisk indicates a significant difference on the five percent level. The boxes indicate the inter quartile range (IQR), the central line depicts the median, the whiskers extend to 1.5*IQR and outliers are plotted individually

For the overall diet repertoire sizes of the adult females and adult males, based on visual examination of the cumulative diet repertoire sizes over increasing follow effort, we included the follow effort as a function of the Michaelis-Menten equation (Lopez et al. 2000). The full model (which included the factor sex and an interaction between sex and follow effort, see Table 2 for model details) fitted the data better than the null model (which only included the random effects; LRT full model versus null model: Chi-square = 3900.3, *P* < 0.001). The full model revealed a significant interaction between the effects of follow effort and sex, and the estimates of the predictors indicated that flanged males overall ate fewer different food items than adult females (Table 2). The difference in overall diet repertoire size between the two sexes became most obvious after 1000 follow hours (Fig. 2). To comparably assess differences in overall diet repertoire sizes between adult females and flanged males, we estimated total diet repertoire sizes of each individual with a function of the Michaelis-Menten equation with a subset of the data, including the full data set (with all available follow hours for each individual) and the first 625 follow hours of each individual (which is the number of total follow hours available on the individual that was followed the least in this analysis). Both analyses indicated that the estimated total diet repertoire sizes of adult females were larger than the ones of flanged males. The analysis with the full data set resulted in a statistically significant result (*T*-test: M_Females_ = 215.710, M_Males_ = 125.982; df = 8.203; t = 4.630, *P* = 0.002, Fig. 3a), and the one with the small subset of the data resulted in a trend (*T*-test: M_Females_ = 142.467, M_Males_ = 111.846; df = 7.357; *t* = 2.207, *P* = 0.061, Fig. 3b). The parameters of the Michaelis-Menten fits for all individuals for each of the two analyses are summarized in Table ESM 5. There were 163 food items which were not consumed by any of the flanged males (but by at least one of the adult females). These food items included all plant item types, honey, wasps, and bird eggs (see “Methods” section). The frequency of these food items in the overall adult females’ diet (assessed via the feeding bouts we had available from the adult female focal animals) ranged from 0.0002 to 0.31 percent (mean = 0.008%, which corresponds to 1–1303 feeding bouts), which is less than the 0.33% average food item frequency across all food items in the adult females’ diet.

**Table 2:** Sex differences in adult overall diet repertoires.

s	Factor type	Estimate	Std-Error	*P*	R^2^
Intercept	Intercept	11.595	2.646	<0.001	0.983
MM (follow effort)	Control	0.926	0.005	**<0.001**	
Sex (m)	Predictor	4.537	3.400	0.182	
Sex (m) * MM (follow effort)	Predictor	−0.218	0.011	**<0.001**	
Individual	Random	-	-	-	

The effects of follow effort and sex on overall diet repertoire sizes of adult females and flanged males analyzed with a GLMM with Gaussian family distribution whereby follow effort was included as a function of the Michaelis-Menten (MM) equation (see Table ESM 5a for details on the Michaelis-Menten fit). R^2^ refers to conditional pseudo delta R^2^. Significant P–values of predictors are indicated with bold font

### Development of diet breadth and complexity in immatures

**Fig. 4:**
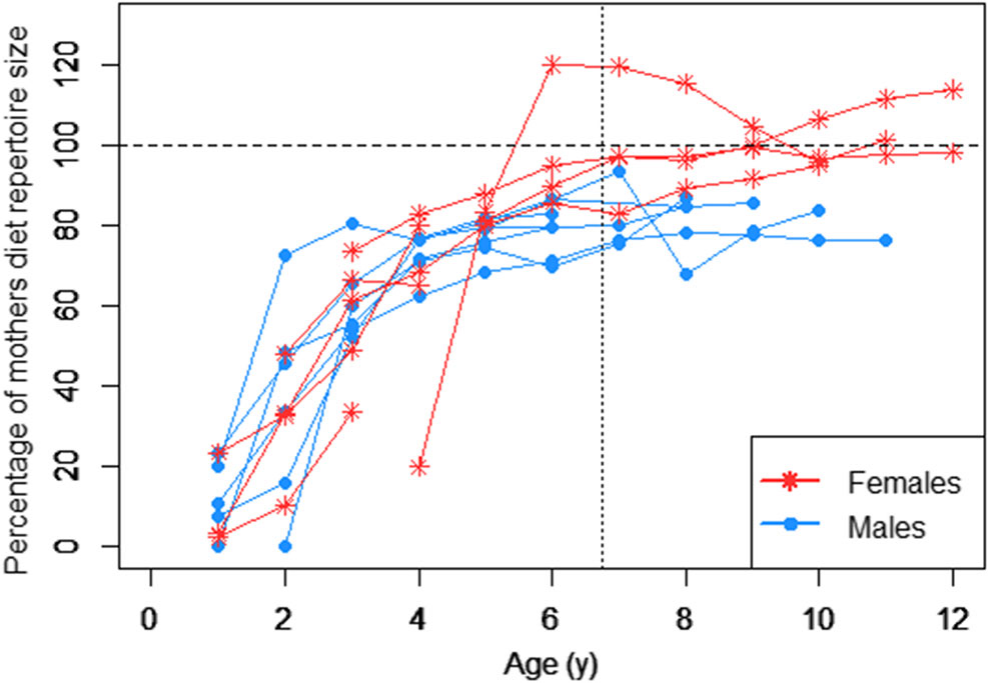
Development of overall diet repertoire size: Diet repertoire size (in percent of the mother’s diet repertoire size within the same time period) over age for different immature. Each line represents one immature individual and the vertical dashed line represents the average weaning age at the population

We found several indications for differences in diet repertoire development in immature males and females. For the overall diet repertoire size, we found that in both sexes, the number of food items in the diet increased with age. However, female immatures seemed to acquire a broader diet faster than male immatures: Females reached similar or even larger diet breadths as their mothers by the age of weaning, whereas males at that point only reached around 80% (Fig. 4). Based on the visual examination of the data, we included age as a sigmoid factor. The full model (which included the predictors sex, sigmoid age, and the interaction between the sigmoid age factor and sex; see Table 3 for model details) fitted the data better than the null model (which included the random effect and control predictor; LRT full model versus null model: Chi-square = 448.280, *P* < 0.001). The full model revealed a significant interaction between age and sex and the estimates of the model indicated that immature females developed broader diets faster than immature males (Fig. 4, Table 3).

**Table 3:** Development of overall diet repertoire size

Factor	Factor type	Estimate	Std-Error	*P*	R^2^	Dispersion parameter
Intercept	Intercept	−10.272	0.870	<0.001	0.960	1.001
Sigmoid (age)	Predictor	10.206	0.884	**<0.001**		
Sex (m)	Predictor	3.545	0.993	**<0.001**		
Sex (m)*sigmoid (age)	Predictor	−3.684	1.012	**<0.001**		
Mothers diet repertoire size	Control (Offset)	-	-	-		
Individual	Random	-	-	-		

The effects of age and sex on immatures’ overall diet repertoire size (i.e., the number of food items in the diet of an individual), analyzed with a GLMM with Poisson family distribution. R^2^ refers to conditional pseudo delta R^2^. Significant *P*–values of predictors are indicated with bold font

**Fig. 5:**
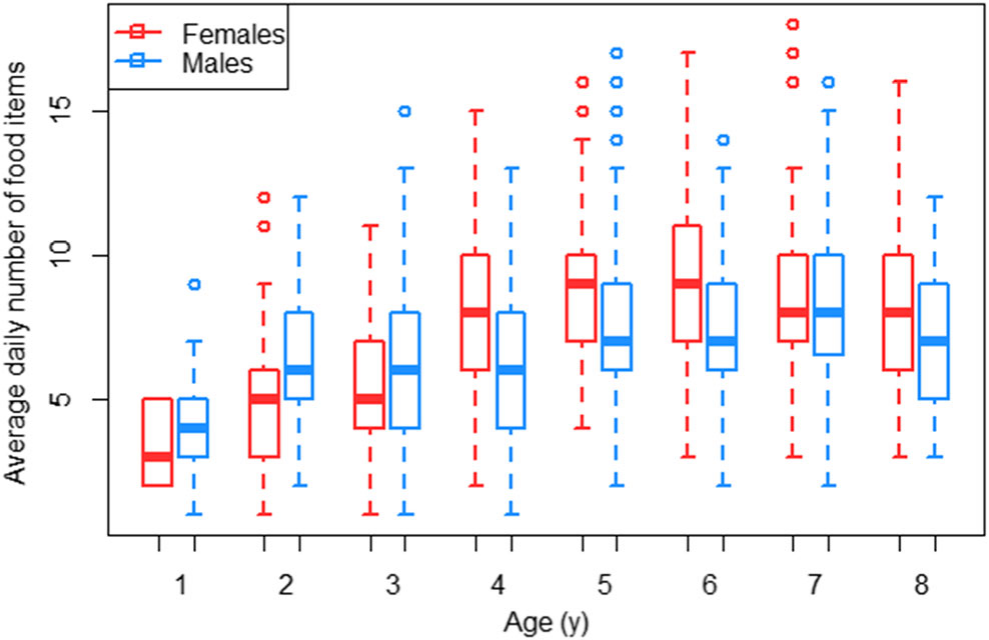
Development of daily diet breadth: Average number of different food items eaten per day by female and male dependent immatures over age. The boxes indicate the inter quartile range (IQR), the central line depicts the median, the whiskers extend to 1.5*IQR and outliers are plotted individually

We found that for both sexes, the daily diet breadth increased with age and reached adult-level values around the age of 5 years. Based on the visual examination of the data, we included age as a sigmoid factor. We found that the full model (which included the predictors sex, sigmoid age, and the interaction between the sigmoid age factor and sex; see Table 4 for model details) fitted the data better than the null model (which only included the random effects; LRT full model versus null model: Chi-square = 121.660, *P* < 0.001). We also found that after age three, the number of daily food items eaten by females increased faster than those eaten by males (Table 4, Fig. 5). However, when comparing the relative overall diet breadth (number of different food items eaten controlled for follow effort, measured during the dependency period of the offspring) of mothers with female offspring to the relative diet breadth of mothers with male offspring, we found that the full model (GLMM_Poisson_: mother’s diet breadth ~offspring sex + follow hours + (1|mother)) did not fit the data better than the null model (which only included the random effect and follow hours; LRT full model versus null model: Chi-square = 0.0074, *P* = 0.9316).

**Table 4:** Development of daily diet breadth

Factor	Factor type	Estimate	Std-Error	*P*	R^2^	Dispersion parameter
Intercept	Intercept	−1.959	0.528	<0.001	0.322	0.685
Sigmoid (age)	Predictor	4.051	0.541	**<0.001**		
Sex(m)	Predictor	1.303	0.623	**0.037**		
Sex(m)*sigmoid (age)	Predictor	-1.430	0.639	**0.025**		
Year month	Random	-	-	-		
Individual	Random	-	-	-		

The effects of age and sex on the number of food items eaten by immatures per day, analyzed with a GLMM with Poisson family distribution. R^2^ refers to conditional pseudo delta R^2^. Significant *P*–values of predictors are indicated with bold font

**Fig. 6:**
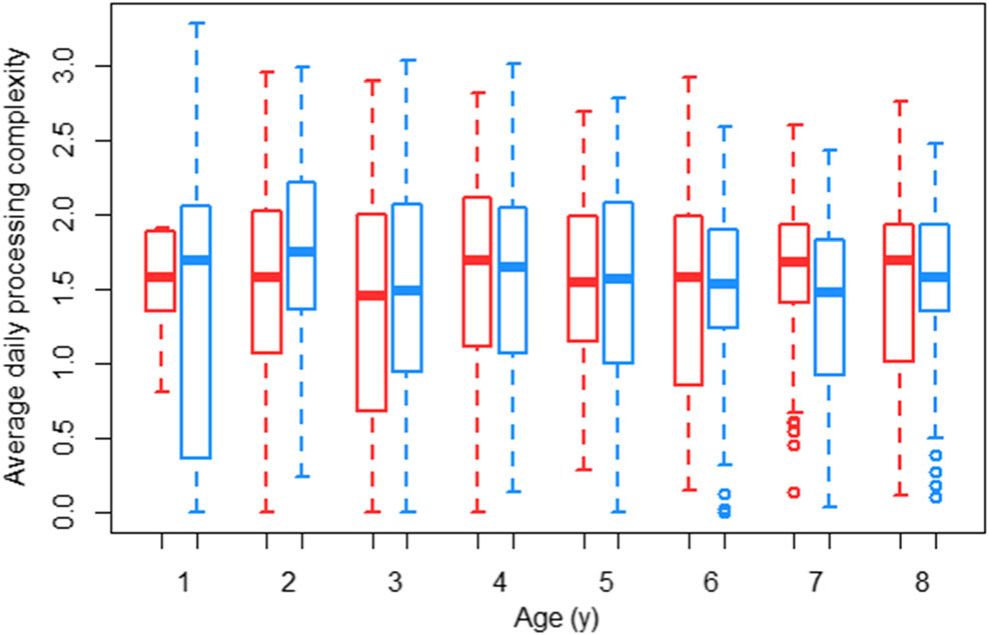
Development of daily diet processing complexity: Average daily diet processing complexity of female and male dependent immatures over age. The boxes indicate the inter quartile range (IQR), the central line depicts the median, the whiskers extend to 1.5*IQR and outliers are plotted individually

**Fig. 7:**
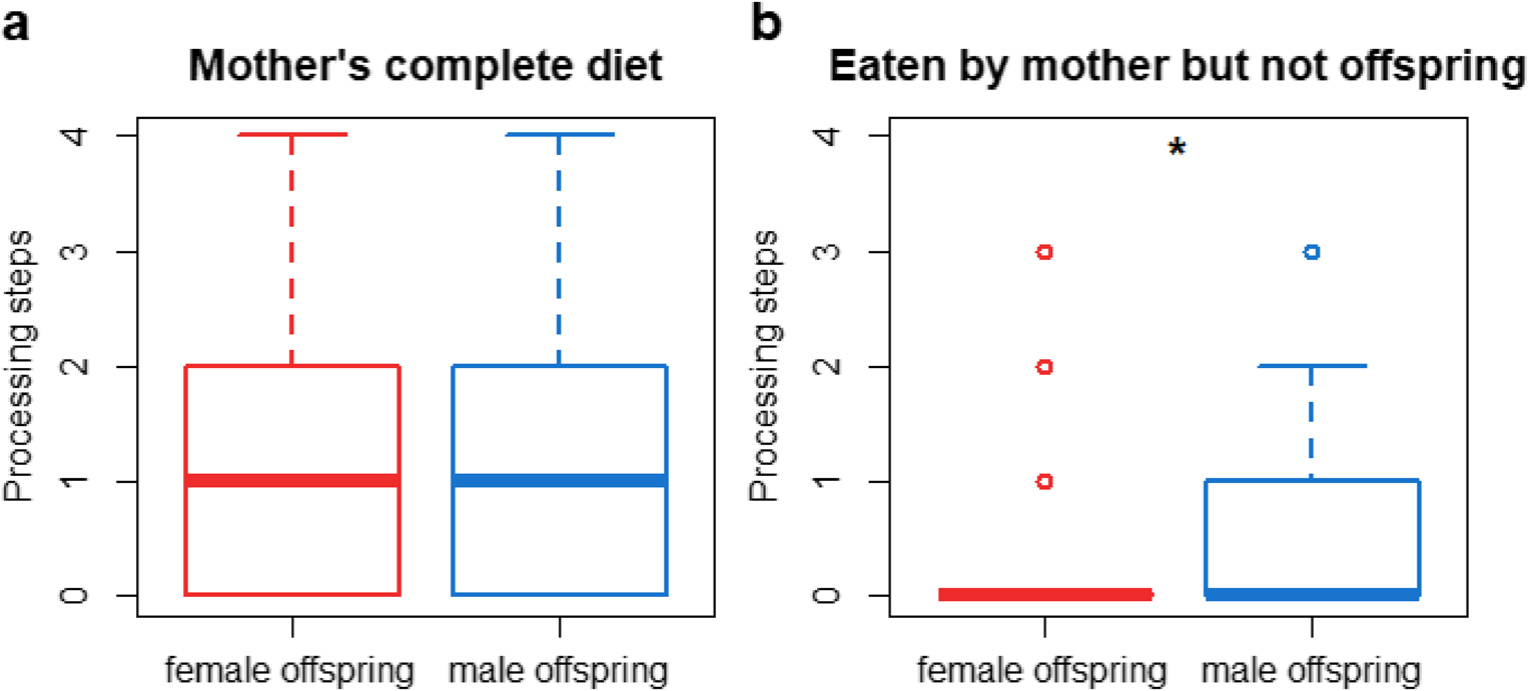
Maternal diet processing complexity: Diet processing complexity of mothers with female and male offspring (**a**) and processing complexity of the food items that are only eaten by the mother but not her offspring for mothers with female and male offspring (**b**). The asterisk indicates a significant difference on the five percent level. The boxes indicate the inter quartile range (IQR), the central line depicts the median, the whiskers extend to 1.5*IQR and outliers are plotted individually

For the development of daily diet processing complexity, we found that the full model (GLMM_Gaussian_: average daily processing complexity ~sex + age + (1|individual)) did not fit the data better than the null model (which only included the random effect; LRT full model versus null model: Chi-square = 0.615, *P* = 0.735, Fig. 6) for the development of daily diet processing complexity of the immatures. When comparing the processing complexity of the diets of mothers with female offspring to that of mothers with male offspring (measured during the dependency period of the offspring) we found that the full model (GLMM_poisson_: processing complexity ~offspring sex + (1|mother offspring pair/mother) did not fit the data better than the null model (which only included the random effect; LRT full model versus null model: Chi-square = 2.889, *P* = 0.236, Fig. 7a). Thus, mothers did not change their diets depending on the sex of their infant.

To investigate this on a more fine-grained level for each mother-offspring pair, we also analyzed the food items that only the mother but not the dependent immature ate. We found that the full model (which included the predictor offspring sex, see table 5 for model details) fitted the model better than the null model (which only included the random effect; LRT full model versus null model: Chi-square = 10.351, *P* = 0.001). The full model revealed that the food items in the mother’s repertoire that were not observed to be eaten by the offspring were more complex for mothers with male offspring than for mothers with female offspring (Table 5, Fig. 7b). The food items in a mother’s diet that were not observed to be eaten by their male offspring accounted for 12–37 food items and varied in their frequency in the respective mother’s feeding data. Those food items accounted for 0.001–4.927% (mean = 0.277) percent of the feeding bouts of the mother of the offspring (which corresponds to 1–1634 feeding bouts, mean = 113.128 during the dependency period of the offspring) whereas the average food item frequency in each mother’s individual diet ranged from 0.53 to 1.63%.

**Table 5:** Complexity of food items in the mothers’ repertoires that were not observed to be eaten by the offspring

Factor	Factor type	Estimate	Std-Error	P	R^2^	Dispersion parameter
Intercept	Intercept	−1.471	0.293	<0.001	0.183	1.707
Offpsring sex(m)	Predictor	1.218	0.317	**0.001**		
Year month	Random	-	-	-		
Mother offspring pair	Random	-	-	-		

The effects of offspring sex on the complexity of food items eaten only by the mother but not the offspring, analyzed with a GLMM with Poisson family distribution. R^2^ refers to conditional pseudo delta R^2^. Significant *P*–values of predictors are indicated with bold font

## Discussion

Our results showed that male and female Bornean orangutans differ in their feeding behavior as adults and that those differences start to develop during the immature period.

We found that the daily diet of adult females was significantly broader and significantly more complex to process than that of flanged males, even though effect sizes were small (Fig. 1a, b). We found the same difference between the sexes in relative diet breadth (in which the number of daily feeding bouts is controlled for, Fig. 1c), which is in line with previous work showing that males have longer feeding patch residence times (van Schaik et al. 2009). These results thus concur with our prediction that females go to greater lengths to achieve broad and thus presumably more balanced diets. Previous studies have shown differences in the macronutrient composition of the overall diet and relative energy return between the two sexes (Knott 1998; Vogel et al. 2017). Taken together, these results provide strong evidence that adult female and flanged male orangutans have different dietary needs and fulfill them by sex-specific foraging strategies and uses of their foraging niche. They are also consistent with findings of previous studies at Tuanan and elsewhere, namely that even though adult females have significantly longer daily active periods than flanged males, they do not spend more time feeding (van Schaik et al. 2009; Vogel et al. 2017).

Differences in daily averages, however, do not permit definitive conclusions about the overall diets of adult females and adult males. It might well be that even though daily averages differ, overall diet repertoires do not. Yet, in accordance with their higher daily diet breadth, the overall diet repertoire sizes of adult females increased faster than those of flanged males. Unfortunately, whereas for the adult females, we have several individuals with around 4000 follow hours, for flanged males the maximum currently lies at around 2000 follow hours. However, the trajectories of the diet repertoires over increasing follow effort suggested a faster flattening and thus lower overall repertoire for flanged males than for females (Fig. 2), which was further supported by estimates of the full repertoire sizes via the Michaelis-Menten equation.

Even though flanged males have larger home ranges than adult females (Mitra Setia et al. 2009; Spillmann et al. 2017), we follow them only where this range overlaps with that of our focal females (i.e., in the study area), and they thus have the same feeding opportunities as these females. Therefore, the difference in repertoire size is not caused by males not encountering these food items. The food items that were eaten by the females but none of the flanged males, are not out of reach for the flanged males (i.e., they do not grow in a part of the trees which the males cannot reach), which suggests that the flanged males would have access to those non-consumed food items. For example, all of the adult female but none of the flanged male focal animals ate the fruits of Akar Darak, a liana which grows in trees throughout the study area and mostly at very well reachable positions. However, notably, the estimates for the size of the overall diet repertoire differed depending on which data set we used (the full data set which included all follow hours versus the reduced data set which included the first 625 follow hours of each individual only): the larger data set leads to higher estimates (Fig. 3 a, b). This suggests that for neither flanged males nor females, we had enough follow hours available to capture their full repertoires. Therefore, despite the evidence that our results provide at the current stage, to fully confirm that flanged males have lower overall repertoire sizes, more follow hours on them are needed. In addition, males might also include items not available within the study area.

In general, sex differences in diet could be the result of divergent energetic needs imposed by body size and ranging patterns and/or the result of differing reproductive requirements (van Schaik et al. 2009). The larger body size in flanged males would favor food items that have high energy return rates per unit processing time, because this reduces costly travel, despite the fact that overall, their energy intake does not differ from adult females (Vogel et al. 2017). Furthermore, because in males reproductive success is dependent on consortship success (Dunkel et al. 2013), there might be a tradeoff between the time devoted to feeding and reproduction. Female reproduction requires the formation of an exceptional amount of new tissue which is most likely supported by essential micronutrients (Demment, 1983). Furthermore, because of their potential harm to the fetus or suckling infant, reproducing females are expected to avoid the accumulation of toxins. Our results that adult females showed more variable and more complex daily diets than flanged males are in line with all these predictions. All in all, our results fit the concepts of energy maximization and time minimization (Schoener 1971; Hixon 1982), in that flanged male orangutans seem to limit the time they spend feeding which likely frees time for them to monitor and pursue females and adult females seem to maximize their intake (van Schaik et al. 2009). However, one would have to directly analyze the intakes of nutrients and toxins, energy returns in relation to processing time, and energy spent during processing to fully confirm these predictions. Furthermore, our approach did not allow us to quantify the time individuals spent searching for food sources, which might entail additional energetic costs and thus might be an aspect in which the sexes differ as well.

Given the adult differences in diet, we examined whether these differences in foraging behavior between the sexes are reflected in immatures’ behavioral development. We found that immature females reached diet repertoires of the sizes of their mothers’ faster than immature males. Furthermore, whereas weanling females (i.e., individuals who are around the age of weaning) seemed to match or even exceed the size of their mothers’ diet repertoire, weanling males seemed to reach stable diet repertoire sizes before weaning, at only around 80% of their mothers’ diet repertoire size (Fig. 4, Table 3). The food items that were not consumed by the male offspring had a low to medium frequency in the mother’s diet, with some of these items eaten during more than 1600 feeding bouts by the mother during the dependency period of the respective offspring. These findings on the immatures support our preliminary conclusion on the sex differences in adult diets. They also provide strong evidence that immatures start showing behavioral adjustments in their feeding behavior at an age when the two sexes are still similar in physical size and growth rates^,^ (Leigh and Shea 1995; Chappell et al. 2015), and thus likely before sex differences become energetically relevant.

The results on the development of overall diet repertoire size also suggest that immature females go through a phase during which their diets are larger than the ones of their mothers. Because learning is never error-free, diet repertoires would deteriorate over generations if female immatures were to only acquire their diets by learning from their mothers. Therefore, this overshoot might be a result of female immatures learning through independent exploration or from indiviudals other than the mother. Because we have no evidence that ourangutan diets increase over time, females might eventually drop some of these food items again.

Investigating how daily diet breadth develops, we found that from the age of four years onwards, immature females showed a larger daily diet breadth than immature males (Fig. 5, Table 4). This finding strengthens our previous results on the same population which were based on a narrower version of the same data set used here (15,230 simultaneous mother offspring follow hours on 6 immatures versus 26,555 simultaneous mother offspring follow hours on 14 immature individuals used here) and were analyzed with a different approach (Schuppli et al. 2016a). The early emergence of this sex difference is remarkable, given that at that age, dependent immatures of both sexes spend 100% of their time in close association with their mothers, rarely meet adult males which could serve as role models, and are not weaned for at least another two years (van Noordwijk et al. 2009).

We found no evidence for a sex difference in the developmental trajectories for daily diet processing complexity (Fig. 6). Furthermore, from as early as one year of age, the average daily diet processing complexity of the immatures was highly comparable to the one of their mothers and subsequently roughly stayed the same across immature development. This is in line with a previous study on the Tuanan population, showing that immatures can process all types of food items starting at a very early age (Dunkel 2006). What remains unclear is if the sexes differ in the efficiency at which they process food items of varying processing complexity at different ages. In chimpanzees (*Pan troglodytes*), immature females reach proficiency in insect tool use, a highly complex foraging behavior, faster than their male peers (Lonsdorf et al. 2004), and this is reflected in the behaviors of their mothers (e.g., tolerance during begging, Estienne et al. 2019).

We found no evidence for differences in diet processing complexity or diet breadth for mothers with female offspring compared to mothers with male offspring, which suggests that mothers do not adjust their diets to their offspring’s sex (Fig. 7a). These results speak against an active role of the mother in shaping the development of sex-specific foraging behavior in orangutans, at least in the aspects investigated here. However, for the food items eaten only by the mother but not by the immatures, we found that the food items lacking from the immature males’ diets tended to be the more complex ones in their mother’s diet, whereas this was not found for immature females (Fig. 7b). This finding suggests that also for diet processing complexity, there might be some degree of sex-specific development, although less pronounced than diet breadth.

All in all, the result that female orangutans have broader and more complex diets than males are similar to patterns found in humans. Women across cultures eat more varied and balanced diets than men (Wardle et al. 2004; Kiefer et al. 2005; Driskell et al. 2006) and these differences become apparent as early as the childhood age (Field et al. 1999). This suggests that despite socio-cultural gender stereotypes, sex-specific diet preferences may be innate or based on innate learning predispositions and thus the result of differing physiological needs between the sexes. Our results on the orangutans suggest that these patterns might be a great ape universal.

In conclusion, we found that adult orangutans differ in several aspects of their feeding behavior, in line with the differing nutritional needs of the sexes (Vogel et al. 2017). Several aspects of these differences become apparent in behavioral development from an early age on, most likely well before they become energetically relevant (i.e., before the sexes begin to differ in size or behavior). At the current stage, it remains unclear to what extent the nature of these sex differences is based on intrinsic behavioral tendencies as opposed to intrinsic or acquired differences in learning strategies.

## Supplementary Information

ESM 1 (DOCX 116 kb)

## Data Availability

The datasets generated during and/or analyzed during the current study can be accessed through Harward Dataverse (10.7910/DVN/ROLRYH).
