# Supplementary material for: The development and maintenance of sex differences in dietary breadth and complexity in Bornean orangutans
Source: Behav Ecol Sociobiol. 2021 Apr 21;75(5):81. doi: 10.1007/s00265-021-03014-3 (PMC8550522; doi:10.1007/s00265-021-03014-3)
Supplement: Supplementary file 1 — (DOCX 116 kb) [file 265_2021_3014_MOESM1_ESM.docx]

**Behavioral Ecology and Sociobiology**

**The development and maintenance of sex differences in dietary breadth and complexity in Bornean orangutans**

Caroline Schuppli^1,2*^, S. Suci Utami Atmoko^3^, Erin R. Vogel^4^, Carel P. van Schaik^5^, Maria A. van Noordwijk^5^

1: Max Planck Institute of Animal Behavior, Konstanz, Germany.

2: Leipzig Research Center for Early Child Development, University of Leipzig, Germany.

3: Fakultas Biologi, Universitas Nasional, Jakarta, Indonesia.

4: Department of Anthropology, Rutgers University, New Brunswick, New Jersey, USA.

5: Department of Anthropology, University of Zürich, Switzerland.

*Corresponding author: [caroline.schuppli@aim.uzh.ch](mailto:caroline.schuppli@aim.uzh.ch); ORCID: 0000-0001-6318-2815

**Electronic Supplementary Material:**


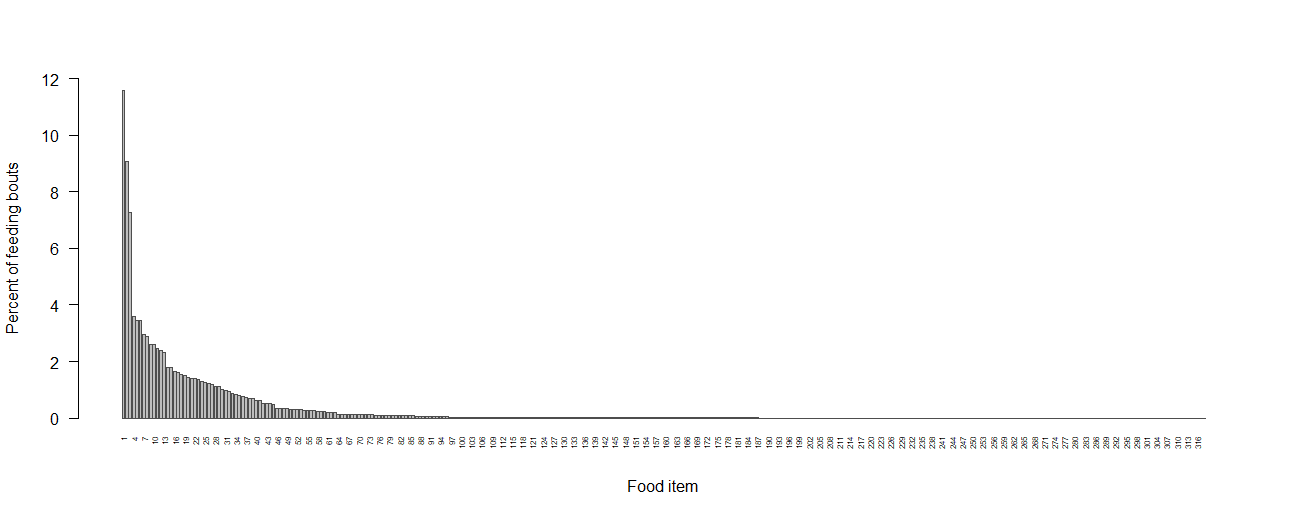


**Fig. ESM 1 Frequency of the different food items:** percentage of feeding bouts the adult focal animals spent feeding on the 318 different food items recorded in the data set of this study (during the 497’720 feeding bouts of the adult focal animals). The average frequency over all food items was 0.31 percent


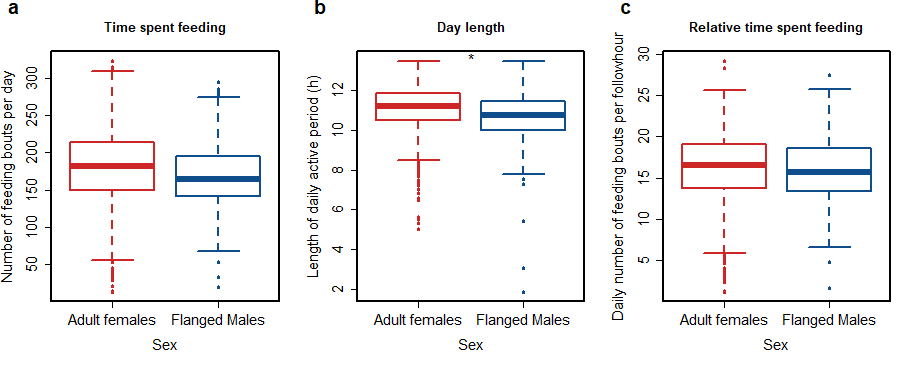


**Fig. ESM 2 Foraging time allocation of adult females and flanged males:** daily number of feeding bouts (a), the length of the daily active period (b) and the daily relative feeding time (number of feeding bouts divided by the length of the active period). The asterisks indicate significant differences on the five percent level

**Table ESM 1 Food items.** Food items in the diets of the focal animals of this study. Number of feeding bouts refers to the number of feeding bouts we recorded adult focal individuals feeding on this item. Population frequency refers to the percentage of adult feeding in which the adult focal individuals fed on the item.

| **Food item** | **Species local name** | **Species latin** | **Item** | **Processing step** | **Number of feeding bouts** | **Population frequency** |
| --- | --- | --- | --- | --- | --- | --- |
| akar balale fr | Akar Balale |  | fruits | NA | 2 | 0.0004 |
| akar buntut ve | Akar Buntut | *Paraphalaenopsis* sp. | vegetative matter | 0 | 34 | 0.0068 |
| akar dangu fl | Akar Dangu | *Willughbeia* sp. | flowers | 0 | 1718 | 0.3438 |
| akar dangu fr | Akar Dangu | *Willughbeia* sp. | fruits | 2 | 11659 | 2.3331 |
| akar dangu lv | Akar Dangu | *Willughbeia* sp. | leaves | 0 | 25 | 0.0050 |
| akar darak fr | Akar Darak | *Desmos* sp. | fruits | 3 | 56 | 0.0112 |
| akar darak lv | Akar Darak | *Desmos* sp. | leaves | 0 | 2 | 0.0004 |
| akar kalalawit fl | Akar Kalalawit | *Uncaria* sp. | flowers | NA | 15 | 0.0030 |
| akar kalalawit fr | Akar Kalalawit | *Uncaria* sp. | fruits | NA | 35 | 0.0070 |
| akar kalalawit lv | Akar Kalalawit | *Uncaria* sp. | leaves | 0 | 1157 | 0.2315 |
| akar kalalawit ve | Akar Kalalawit | *Uncaria* sp. | vegetative matter | NA | 22 | 0.0044 |
| akar kambalitan bk | Akar Kambalitan |  | bark | NA | 55 | 0.0110 |
| akar kambalitan fl | Akar Kambalitan |  | flowers | 0 | 12 | 0.0024 |
| akar kambalitan fr | Akar Kambalitan |  | fruits | 3 | 3020 | 0.6043 |
| akar kambalitan lv | Akar Kambalitan |  | leaves | 0 | 22 | 0.0044 |
| akar kamunda fl | Akar Kamunda | *Leucomphalos callicarpus* | flowers | 0 | 17230 | 3.4479 |
| akar kamunda fr | Akar Kamunda | *Leucomphalos callicarpus* | fruits | 2 | 45302 | 9.0655 |
| akar kamunda lv | Akar Kamunda | *Leucomphalos callicarpus* | leaves | 0 | 57844 | 11.5753 |
| akar kamunda ve | Akar Kamunda | *Leucomphalos callicarpus* | vegetative matter | 0 | 52 | 0.0104 |
| akar katukatu lv | Akar Katukatu |  | leaves | 0 | 1 | 0.0002 |
| akar kecil fl | Akar Kecil |  | flowers | 0 | 14 | 0.0028 |
| akar kecil fr | Akar Kecil |  | fruits | NA | 181 | 0.0362 |
| akar kecil ve | Akar Kecil |  | vegetative matter | 1 | 8264 | 1.6537 |
| akar kelakai lv | Akar Kelakai | *Stenochlaena palustris* | leaves | 0 | 89 | 0.0178 |
| akar kelakai pi | Akar Kelakai | *Stenochlaena palustris* | pith | 1 | 79 | 0.0158 |
| akar kelakai ve | Akar Kelakai | *Stenochlaena palustris* | vegetative matter | NA | 6 | 0.0012 |
| akar kelanis fl | Akar Kelanis | *Alyxia* sp. | flowers | NA | 17 | 0.0034 |
| akar kelanis lv | Akar Kelanis | *Alyxia* sp. | leaves | 0 | 1283 | 0.2567 |
| akar kelanis ve | Akar Kelanis | *Alyxia* sp. | vegetative matter | NA | 73 | 0.0146 |
| akar kuku elang fl | Akar Kuku Elang |  | flowers | NA | 15 | 0.0030 |
| akar kuku elang fr | Akar Kuku Elang |  | fruits | 1 | 717 | 0.1435 |
| akar kuku elang lv | Akar Kuku Elang |  | leaves | NA | 2 | 0.0004 |
| akar kuning fr | Akar Kuning | *Fibraurea tinctoria* | fruits | 2 | 3901 | 0.7806 |
| akar kuning lv | Akar Kuning | *Fibraurea tinctoria* | leaves | 0 | 14 | 0.0028 |
| akar kuning ve | Akar Kuning | *Fibraurea tinctoria* | vegetative matter | NA | 1 | 0.0002 |
| akar lakum lv | Akar Lakum |  | leaves | 0 | 11 | 0.0022 |
| akar pari-pari fr | Akar Pari-Pari | *Momordica* sp. | fruits | 3 | 189 | 0.0378 |
| akar pasike fr | Akar Pasike |  | fruits | NA | 6 | 0.0012 |
| akar pinang pinang fr | Akar Pinang Pinang |  | fruits | 0 | 3465 | 0.6934 |
| akar sarang burung ve | Akar Sarang Burung |  | vegetative matter | NA | 8 | 0.0016 |
| akar takapal fr | Akar Takapal | *Hoya* sp. | fruits | NA | 1 | 0.0002 |
| akar takapal lv | Akar Takapal | *Hoya* sp. | leaves | 0 | 103 | 0.0206 |
| akar takapal ve | Akar Takapal | *Hoya* sp. | vegetative matter | NA | 30 | 0.0060 |
| akar tampelas fr | Akar Tampelas | *Gnetum* sp. | fruits | 1 | 423 | 0.0846 |
| akar tampelas lv | Akar Tampelas | *Gnetum* sp. | leaves | 0 | 10 | 0.0020 |
| akar tampelas ve | Akar Tampelas | *Gnetum* sp. | vegetative matter | 0 | 13 | 0.0026 |
| akar tataji fr | Akar Tataji |  | fruits | NA | 4 | 0.0008 |
| akar tungkun fl | Akar Tungkun |  | flowers | 0 | 10 | 0.0020 |
| akar tungkun fr | Akar Tungkun |  | fruits | 1 | 8 | 0.0016 |
| akar unak fr | Akar Unak |  | fruits | NA | 14 | 0.0028 |
| akar uwei nyamei fr | Akar Uwei Nyamei | *Flagellaria indica* | fruits | 0 | 1617 | 0.3236 |
| akar uwei nyamei lv | Akar Uwei Nyamei | *Flagellaria indica* | leaves | 0 | 4 | 0.0008 |
| akar uwei nyamei ve | Akar Uwei Nyamei | *Flagellaria indica* | vegetative matter | NA | 43 | 0.0086 |
| anggrek fr | Anggrek |  | fruits | NA | 4 | 0.0008 |
| anggrek lv | Anggrek |  | leaves | 0 | 122 | 0.0244 |
| anggrek pi | Anggrek |  | pith | NA | 2 | 0.0004 |
| anggrek ve | Anggrek |  | vegetative matter | NA | 301 | 0.0602 |
| balawan fr | Balawan | *Tristaniopsis* sp. | fruits | NA | 33 | 0.0066 |
| balawan lv | Balawan | *Tristaniopsis* sp. | leaves | 0 | 2 | 0.0004 |
| bengaris bk | Bengaris | *Koompassia malaccensis* | bark | 3 | 318 | 0.0636 |
| bengaris lv | Bengaris | *Koompassia malaccensis* | leaves | 0 | 5 | 0.0010 |
| bengaris ve | Bengaris | *Koompassia malaccensis* | vegetative matter | NA | 5 | 0.0010 |
| bintan fl | Bintan | *Licania splendens* | flowers | NA | 16 | 0.0032 |
| bintan fr | Bintan | *Licania splendens* | fruits | 0 | 181 | 0.0362 |
| damon fr | Damon | *Xerospermum noronhianum* | fruits | 3 | 352 | 0.0704 |
| enyak beruk fl | Enyak Beruk | *Syzygium* sp. | flowers | NA | 11 | 0.0022 |
| enyak beruk fr | Enyak Beruk | *Syzygium* sp. | fruits | 2 | 4874 | 0.9753 |
| enyak beruk lv | Enyak Beruk | *Syzygium* sp. | leaves | 0 | 5 | 0.0010 |
| epiphyte lv | Epiphyte |  | leaves | 0 | 28 | 0.0056 |
| epiphyte ve | Epiphyte |  | vegetative matter | NA | 51 | 0.0102 |
| fungi ot | Fungi |  | other | 0 | 1 | 0.0002 |
| galaga fr | Galaga | *Santiria/Dacryodes* sp. | fruits | NA | 526 | 0.1053 |
| galam tikus fl | Galam Tikus | *Syzygium curtisii* | flowers | 0 | 52 | 0.0104 |
| galam tikus fr | Galam Tikus | *Syzygium curtisii* | fruits | 0 | 3531 | 0.7066 |
| galam tikus lv | Galam Tikus | *Syzygium curtisii* | leaves | 0 | 5 | 0.0010 |
| galam tikus ve | Galam Tikus | *Syzygium curtisii* | vegetative matter | NA | 2 | 0.0004 |
| gandis fr | Gandis | *Garcinia cf. parviflora* | fruits | 2 | 152 | 0.0304 |
| gandis lv | Gandis | *Garcinia cf. parviflora* | leaves | 0 | 8 | 0.0016 |
| gerising besar fl | Gerising Besar | *Pandanus* sp. | flowers | NA | 1 | 0.0002 |
| gerising besar fr | Gerising Besar | *Pandanus* sp. | fruits | 1 | 1558 | 0.3118 |
| gerising besar lv | Gerising Besar | *Pandanus* sp. | leaves | 0 | 9 | 0.0018 |
| gerising besar ve | Gerising Besar | *Pandanus* sp. | vegetative matter | 1 | 1482 | 0.2966 |
| gerising kecil fl | Gerising Kecil | *cf. Freycinetia* | flowers | NA | 3 | 0.0006 |
| gerising kecil fr | Gerising Kecil | *cf. Freycinetia* | fruits | 1 | 13 | 0.0026 |
| gerising kecil ve | Gerising Kecil | *cf. Freycinetia* | vegetative matter | 1 | 79 | 0.0158 |
| gerising kelep fr | Gerising Kelep |  | fruits | NA | 9 | 0.0018 |
| hampuak lv | Hampuak | *Euphorbiaceae* | leaves | 0 | 2 | 0.0004 |
| hangkang fr | Hangkang | *Pallaquium leiocarpum* | fruits | 2 | 11939 | 2.3891 |
| hangkang lv | Hangkang | *Pallaquium leiocarpum* | leaves | 0 | 2 | 0.0004 |
| hangkang ve | Hangkang | *Pallaquium leiocarpum* | vegetative matter | NA | 2 | 0.0004 |
| hanyer bajai fr | Hanyer Bajai |  | fruits | 3 | 194 | 0.0388 |
| hitam hitam fr | Hitam Hitam |  | fruits | 0 | 1056 | 0.2113 |
| hitam hitam lv | Hitam Hitam |  | leaves | 0 | 2 | 0.0004 |
| bees ho | Honey |  | insects | 3 | 32 | 0.0064 |
| kajalaki fr | Kajalaki | *Notophoebe umbeliflora* | fruits | NA | 11 | 0.0022 |
| kajalaki hatue fr | Kajalaki Hatue | *Aglaia* sp. | fruits | 2 | 176 | 0.0352 |
| kajalaki lv | Kajalaki | *Notophoebe umbeliflora* | leaves | 0 | 3 | 0.0006 |
| kambalitan bawi fl | Kambalitan Bawi |  | flowers | NA | 70 | 0.0140 |
| kambalitan bawi fr | Kambalitan Bawi |  | fruits | NA | 4020 | 0.8045 |
| kambalitan bawi lv | Kambalitan Bawi |  | leaves | 0 | 34 | 0.0068 |
| kambasira bawi fr | Kambasira Bawi | *Ilex* sp. | fruits | 0 | 200 | 0.0400 |
| kambasira hatue fr | Kambasira Hatue | *Ilex* sp. | fruits | 0 | 555 | 0.1111 |
| kambasira hatue lv | Kambasira Hatue | *Ilex* sp. | leaves | 0 | 4 | 0.0008 |
| kambasulan lv | Kambasulan | *Rhodamnia cinerea* | leaves | 0 | 1 | 0.0002 |
| kamehas daun kecil fl | Kamehas Daun Kecil |  | flowers | NA | 20 | 0.0040 |
| kamehas daun kecil fr | Kamehas Daun Kecil |  | fruits | 0 | 685 | 0.1371 |
| kamehas daun perak fr | Kamehas Daun Perak | *Cryprtocarya* sp. | fruits | 0 | 108 | 0.0216 |
| kamehas daun perak lv | Kamehas Daun Perak | *Cryprtocarya* sp. | leaves | 0 | 13 | 0.0026 |
| kamuning fl | Kamuning | *Xanthophyllum ecarinatum* | flowers | 0 | 20 | 0.0040 |
| kamuning fr | Kamuning | *Xanthophyllum ecarinatum* | fruits | NA | 34 | 0.0068 |
| kamuning lv | Kamuning | *Xanthophyllum ecarinatum* | leaves | 0 | 6430 | 1.2867 |
| kamuning ve | Kamuning | *Xanthophyllum ecarinatum* | vegetative matter | NA | 1 | 0.0002 |
| kandurin lv | Kandurin | *Gluta* sp. | leaves | 0 | 4 | 0.0008 |
| kapurnaga jangkar fr | Kapurnaga Jangkar |  | fruits | 3 | 11 | 0.0022 |
| kapurnaga jangkar lv | Kapurnaga Jangkar |  | leaves | 0 | 1 | 0.0002 |
| kapurnaga jankar fr | Kapurnaga Jankar | *Calophyllum inophyllum* | fruits | 3 | 483 | 0.0967 |
| kapurnaga jankar lv | Kapurnaga Jankar | *Calophyllum inophyllum* | leaves | 0 | 79 | 0.0158 |
| karamuan fr | Karamuan | *Alseadaphne* sp. | fruits | NA | 221 | 0.0442 |
| karamuan lv | Karamuan | *Alseadaphne* sp. | leaves | 0 | 17 | 0.0034 |
| karamunting fr | Karamunting | *Melastoma malabahricum* | fruits | NA | 2 | 0.0004 |
| karandau putih fl | Karandau Putih | *Blumeadendron kurzii* | flowers | NA | 21 | 0.0042 |
| karandau putih fr | Karandau Putih | *Blumeadendron kurzii* | fruits | 3 | 6976 | 1.3960 |
| karandau putih lv | Karandau Putih | *Blumeadendron kurzii* | leaves | 0 | 57 | 0.0114 |
| katiau bk | Katiau | *Madhuca motleyana* | bark | 3 | 1 | 0.0002 |
| katiau fl | Katiau | *Madhuca motleyana* | flowers | 0 | 14366 | 2.8748 |
| katiau fr | Katiau | *Madhuca motleyana* | fruits | 1 | 962 | 0.1925 |
| katiau lv | Katiau | *Madhuca motleyana* | leaves | 0 | 58 | 0.0116 |
| kayu lalas lv | Kayu Lalas | *Musaendopsis beccariana* | leaves | 0 | 4 | 0.0008 |
| kayu sapat fr | Kayu Sapat | *Santiria laevigata* | fruits | 2 | 1141 | 0.2283 |
| kayu sapat lv | Kayu Sapat | *Santiria laevigata* | leaves | 0 | 2 | 0.0004 |
| kayu taji fr | Kayu Taji |  | fruits | NA | 50 | 0.0100 |
| kayu tulang fl | Kayu Tulang | *Diospyros confertifolia* | flowers | 0 | 2 | 0.0004 |
| kayu tulang fr | Kayu Tulang | *Diospyros confertifolia* | fruits | 2 | 653 | 0.1307 |
| keput bajuku fl | Keput Bajuku | *Stemonurus scorpioides* | flowers | NA | 23 | 0.0046 |
| keput bajuku fr | Keput Bajuku | *Stemonurus scorpioides* | fruits | 1 | 11 | 0.0022 |
| keput bajuku lv | Keput Bajuku | *Stemonurus scorpioides* | leaves | 0 | 3 | 0.0006 |
| keranji fl | Keranji | *Dialum indum* | flowers | NA | 2 | 0.0004 |
| keranji lv | Keranji | *Dialum indum* | leaves | 0 | 425 | 0.0850 |
| kumpang daun bulut fr | Kumpang Daun Bulut | *Gymnacranthera farquhariana* | fruits | 3 | 70 | 0.0140 |
| kumpang daun hijau fr | Kumpang Daun Hijau |  | fruits | 3 | 69 | 0.0138 |
| kumpang daun hijau lv | Kumpang Daun Hijau |  | leaves | 0 | 2 | 0.0004 |
| kumpang daun perak fl | Kumpang Daun Perak | *Horsfieldia crassifolia* | flowers | NA | 3 | 0.0006 |
| kumpang daun perak fr | Kumpang Daun Perak | *Horsfieldia crassifolia* | fruits | 3 | 307 | 0.0614 |
| lampesau ve | Lampesau |  | vegetative matter | 1 | 31 | 0.0062 |
| langset kalawet fr | Langset Kalawet | *Santiria* sp. | fruits | 2 | 7 | 0.0014 |
| lewang bk | Lewang | *Pouteria cf malaccensis* | bark | 3 | 2 | 0.0004 |
| lewang fl | Lewang | *Pouteria cf malaccensis* | flowers | 0 | 441 | 0.0882 |
| lewang fr | Lewang | *Pouteria cf malaccensis* | fruits | 2 | 14823 | 2.9663 |
| lewang lv | Lewang | *Pouteria cf malaccensis* | leaves | 0 | 302 | 0.0604 |
| lunuk besar fr | Lunuk Besar | *Ficus sundaica* | fruits | 0 | 5877 | 1.1761 |
| lunuk besar lv | Lunuk Besar | *Ficus sundaica* | leaves | 0 | 2 | 0.0004 |
| lunuk fl | Lunuk | *Ficus* sp. | flowers | NA | 8 | 0.0016 |
| lunuk fr | Lunuk | *Ficus* sp. | fruits | 0 | 8098 | 1.6205 |
| lunuk handipe lv | Lunuk Handipe |  | leaves | 0 | 12 | 0.0024 |
| lunuk kecil fr | Lunuk Kecil | *Ficus* sp. | fruits | 0 | 4191 | 0.8387 |
| lunuk kecil lv | Lunuk Kecil | *Ficus* sp. | leaves | 0 | 38 | 0.0076 |
| lunuk kuning fr | Lunuk Kuning | *Ficus stupenda* | fruits | NA | 8 | 0.0016 |
| lunuk lv | Lunuk | *Ficus* sp. | leaves | 0 | 141 | 0.0282 |
| lunuk merah fr | Lunuk Merah | *Ficus* sp. | fruits | NA | 87 | 0.0174 |
| lunuk tanah fr | Lunuk Tanah | *Ficus* sp. | fruits | NA | 40 | 0.0080 |
| lunuk tanah lv | Lunuk Tanah | *Ficus* sp. | leaves | 0 | 253 | 0.0506 |
| madang fr | Madang |  | fruits | NA | 248 | 0.0496 |
| madang rambut merah fr | Madang Rambut Merah | *Ctenolophon parviflorus* | fruits | 1 | 2460 | 0.4923 |
| mahandingan fl | Mahandingan | *Calophylum nodusum* | flowers | NA | 6 | 0.0012 |
| mahandingan fr | Mahandingan | *Calophylum nodusum* | fruits | 1 | 1355 | 0.2712 |
| mahawai 2 bk | Mahawai 2 | *Polyalthia hypoleuca.* | bark | 3 | 4 | 0.0008 |
| mahawai 2 fr | Mahawai 2 | *Polyalthia hypoleuca.* | fruits | 2 | 6747 | 1.3502 |
| mahawai 2 lv | Mahawai 2 | *Polyalthia hypoleuca.* | leaves | 0 | 2 | 0.0004 |
| mahawai umb bk | Mahawai Umb | *Mezzettia cf. Leptopoda/parviflora* | bark | 3 | 3 | 0.0006 |
| mahawai umb fl | Mahawai Umb | *Mezzettia cf. Leptopoda/parviflora* | flowers | 0 | 34 | 0.0068 |
| mahawai umb fr | Mahawai Umb | *Mezzettia cf. Leptopoda/parviflora* | fruits | 2 | 8995 | 1.8000 |
| mahawai umb lv | Mahawai Umb | *Mezzettia cf. Leptopoda/parviflora* | leaves | 0 | 24 | 0.0048 |
| mangis hutan daun besar fr | Mangis Hutan Daun Besar | *Garcinia cf. Beccarii* | fruits | 3 | 4740 | 0.9485 |
| mangis hutan daun besar lv | Mangis Hutan Daun Besar | *Garcinia cf. Beccarii* | leaves | 0 | 23 | 0.0046 |
| mangis hutan daun kecil fl | Mangis Hutan Daun Kecil | *Garcinia bancana* | flowers | NA | 12 | 0.0024 |
| mangis hutan daun kecil fr | Mangis Hutan Daun Kecil | *Garcinia bancana* | fruits | 3 | 17919 | 3.5858 |
| mangis hutan daun kecil lv | Mangis Hutan Daun Kecil | *Garcinia bancana* | leaves | 0 | 550 | 0.1101 |
| mangkinang blawau fl | Mangkinang Blawau | *Elaeocarpus* sp. | flowers | 0 | 506 | 0.1013 |
| mangkinang blawau fr | Mangkinang Blawau | *Elaeocarpus* sp. | fruits | 1 | 3181 | 0.6366 |
| mangkinang blawau lv | Mangkinang Blawau | *Elaeocarpus* sp. | leaves | 0 | 15 | 0.0030 |
| maranti fl | Maranti | *Shorea parvistipulata* | flowers | NA | 5 | 0.0010 |
| maranti fr | Maranti | *Shorea parvistipulata* | fruits | NA | 18 | 0.0036 |
| maranti lv | Maranti | *Shorea parvistipulata* | leaves | 0 | 10 | 0.0020 |
| mariuh fl | Mariuh |  | flowers | NA | 4 | 0.0008 |
| mariuh fr | Mariuh |  | fruits | NA | 19 | 0.0038 |
| martibu fl | Martibu | *Dactylocladus stenostachys* | flowers | NA | 4 | 0.0008 |
| martibu fr | Martibu | *Dactylocladus stenostachys* | fruits | NA | 1 | 0.0002 |
| maruang bk | Maruang | *Myristica lowianan.* | bark | 3 | 5517 | 1.1040 |
| maruang fl | Maruang | *Myristica lowianan.* | flowers | NA | 1 | 0.0002 |
| maruang fr | Maruang | *Myristica lowianan.* | fruits | 3 | 162 | 0.0324 |
| maruang lv | Maruang | *Myristica lowianan.* | leaves | 0 | 43 | 0.0086 |
| matahari fl | Matahari | *Lepisanthes amoena* | flowers | NA | 2 | 0.0004 |
| mipa lv | Mipa | *Cratoxylum glaucum* | leaves | 0 | 11 | 0.0022 |
| nangka bk | Nangka |  | bark | NA | 30 | 0.0060 |
| nangka fr | Nangka |  | fruits | NA | 426 | 0.0852 |
| nonang fr | Nonang | *Antidesma cf. Cuspidatum* | fruits | 0 | 6 | 0.0012 |
| nyatoh puntik fl | Nyatoh Puntik | *Palaquium pseudorostrum* | flowers | 0 | 7553 | 1.5114 |
| nyatoh puntik fr | Nyatoh Puntik | *Palaquium pseudorostrum* | fruits | 2 | 3676 | 0.7356 |
| nyatoh puntik lv | Nyatoh Puntik | *Palaquium pseudorostrum* | leaves | 0 | 41 | 0.0082 |
| nyatoh puntik pi | Nyatoh Puntik | *Palaquium pseudorostrum* | pith | NA | 2 | 0.0004 |
| nyatoh puntik ve | Nyatoh Puntik | *Palaquium pseudorostrum* | vegetative matter | NA | 3 | 0.0006 |
| nyatoh undus bua merah bk | Nyatoh Undus Bua Merah |  | bark | 3 | 15 | 0.0030 |
| nyatoh undus bua merah fl | Nyatoh Undus Bua Merah |  | flowers | NA | 2 | 0.0004 |
| nyatoh undus buah besar bk | Nyatoh Undus Buah Besar | *Palaquium cochlearifolium* | bark | 3 | 9 | 0.0018 |
| nyatoh undus buah besar fl | Nyatoh Undus Buah Besar | *Palaquium cochlearifolium* | flowers | 1 | 6926 | 1.3860 |
| nyatoh undus buah besar fr | Nyatoh Undus Buah Besar | *Palaquium cochlearifolium* | fruits | 1 | 6306 | 1.2619 |
| nyatoh undus buah besar lv | Nyatoh Undus Buah Besar | *Palaquium cochlearifolium* | leaves | 0 | 38 | 0.0076 |
| nyatoh undus buah merah bk | Nyatoh Undus Buah Merah | *Palaquium ridleyi* | bark | 3 | 3 | 0.0006 |
| nyatoh undus buah merah fl | Nyatoh Undus Buah Merah | *Palaquium ridleyi* | flowers | 0 | 563 | 0.1127 |
| nyatoh undus buah merah fr | Nyatoh Undus Buah Merah | *Palaquium ridleyi* | fruits | 1 | 13070 | 2.6155 |
| nyatoh undus buah merah lv | Nyatoh Undus Buah Merah | *Palaquium ridleyi* | leaves | 0 | 135 | 0.0270 |
| nyatoh undus daun ujung fl | Nyatoh Undus Daun Ujung | *Payena leerii* | flowers | 0 | 26 | 0.0052 |
| nyatoh undus daun ujung fr | Nyatoh Undus Daun Ujung | *Payena leerii* | fruits | 2 | 636 | 0.1273 |
| nyatoh undus daun ujung lv | Nyatoh Undus Daun Ujung | *Payena leerii* | leaves | 0 | 13 | 0.0026 |
| pahawas bk | Pahawas | *Litsea* sp. | bark | NA | 5 | 0.0010 |
| pahawas fl | Pahawas | *Litsea* sp. | flowers | NA | 7 | 0.0014 |
| pahawas fr | Pahawas | *Litsea* sp. | fruits | NA | 10 | 0.0020 |
| pakan buah besar fr | Pakan Buah Besar |  | fruits | 2 | 1611 | 0.3224 |
| pakan buah besar lv | Pakan Buah Besar |  | leaves | 0 | 2 | 0.0004 |
| pakan fl | Pakan | *Parartocarpus* sp. | flowers | 0 | 480 | 0.0961 |
| pakan fr | Pakan | *Parartocarpus* sp. | fruits | 2 | 1399 | 0.2800 |
| pakan lv | Pakan | *Parartocarpus* sp. | leaves | 0 | 3 | 0.0006 |
| paku pakuan lv | Paku Pakuan |  | leaves | 0 | 6 | 0.0012 |
| paku pakuan ve | Paku Pakuan |  | vegetative matter | NA | 5 | 0.0010 |
| pampaning fr | Pampaning | *Lithocarpus conocarpus* | fruits | 3 | 475 | 0.0951 |
| pantung bk | Pantung | *Dyera lowii* | bark | 3 | 7143 | 1.4294 |
| pantung fl | Pantung | *Dyera lowii* | flowers | NA | 5 | 0.0010 |
| pantung fr | Pantung | *Dyera lowii* | fruits | 3 | 5552 | 1.1110 |
| pantung lv | Pantung | *Dyera lowii* | leaves | 0 | 20 | 0.0040 |
| pantung pi | Pantung | *Dyera lowii* | pith | 4 | 12338 | 2.4690 |
| papar buwu fr | Papar Buwu | *Carallia* sp. | fruits | NA | 41 | 0.0082 |
| papung fr | Papung | *Sandoricum borneense* | fruits | 2 | 6142 | 1.2291 |
| papung lv | Papung | *Sandoricum borneense* | leaves | 0 | 2 | 0.0004 |
| pendo fl | Pendo | *Sterculia* sp. | flowers | 0 | 24 | 0.0048 |
| pendo fr | Pendo | *Sterculia* sp. | fruits | NA | 41 | 0.0082 |
| pendo lv | Pendo | *Sterculia* sp. | leaves | 0 | 413 | 0.0826 |
| pendo ve | Pendo | *Sterculia* sp. | vegetative matter | NA | 16 | 0.0032 |
| piais fr | Piais | *Nephelium mangayi* | fruits | 2 | 1303 | 0.2607 |
| piais lv | Piais | *Nephelium mangayi* | leaves | 0 | 22 | 0.0044 |
| pinang bahandang fr | Pinang Bahandang | *Cyrtostachys cf. rendra* | fruits | 3 | 3 | 0.0006 |
| pinang bahandang ve | Pinang Bahandang | *Cyrtostachys cf. rendra* | vegetative matter | NA | 13 | 0.0026 |
| pinang fr | Pinang |  | fruits | 0 | 76 | 0.0152 |
| pinang ve | Pinang |  | vegetative matter | NA | 3 | 0.0006 |
| pinding pandan bk | Pinding Pandan | *Diospyros siamang* | bark | 3 | 380 | 0.0760 |
| pinding pandan fl | Pinding Pandan | *Diospyros siamang* | flowers | 0 | 23 | 0.0046 |
| pinding pandan fr | Pinding Pandan | *Diospyros siamang* | fruits | 3 | 8903 | 1.7816 |
| pinding pandan lv | Pinding Pandan | *Diospyros siamang* | leaves | 0 | 1489 | 0.2980 |
| Pinding pandang fr | Pinding Pandang |  | fruits | NA | 175 | 0.0350 |
| purun tikus ve | Purun Tikus | *flower/grass bare soil* | vegetative matter | 0 | 2 | 0.0004 |
| rahanjang batu fr | Rahanjang Batu | *Xylopia* sp. | fruits | 2 | 20 | 0.0040 |
| rahanjang bawi bk | Rahanjang Bawi |  | bark | 3 | 11 | 0.0022 |
| rahanjang bawi fl | Rahanjang Bawi |  | flowers | 0 | 43 | 0.0086 |
| rahanjang bawi fr | Rahanjang Bawi |  | fruits | 2 | 12952 | 2.5919 |
| rahanjang bawi lv | Rahanjang Bawi |  | leaves | 0 | 6 | 0.0012 |
| rahanjang hatue fr | Rahanjang Hatue | *Xylopia* sp. | fruits | 2 | 69 | 0.0138 |
| rambangon fl | Rambangon | *Acronychia pedunculata* | flowers | NA | 48 | 0.0096 |
| rambangon fr | Rambangon | *Acronychia pedunculata* | fruits | NA | 14 | 0.0028 |
| rambangon lv | Rambangon | *Acronychia pedunculata* | leaves | 0 | 13 | 0.0026 |
| rambutan hutan fr | Rambutan Hutan | *Nephelium* sp. | fruits | 2 | 2591 | 0.5185 |
| rambutan hutan lv | Rambutan Hutan | *Nephelium* sp. | leaves | 0 | 10 | 0.0020 |
| rasau kelep fr | Rasau Kelep |  | fruits | 2 | 2 | 0.0004 |
| rayap in | Rayap |  | insects | 2 | 17192 | 3.4403 |
| rengas parei fr | Rengas Parei | *Buchanania* sp. | fruits | 0 | 543 | 0.1087 |
| rewui fr | Rewui | *Mircocos* sp. | fruits | 1 | 623 | 0.1247 |
| rewui lv | Rewui | *Mircocos* sp. | leaves | 0 | 1 | 0.0002 |
| rotan dahanen ve | Rotan Dahanen | *Plectocomiopsis* | vegetative matter | NA | 2 | 0.0004 |
| rotan fr | Rotan |  | fruits | 2 | 133 | 0.0266 |
| rotan nyamei pi | Rotan Nyamei |  | pith | NA | 2 | 0.0004 |
| rotan ve | Rotan |  | vegetative matter | 3 | 74 | 0.0148 |
| ruas fr | Ruas |  | fruits | NA | 61 | 0.0122 |
| san kuwuk ve | San Kuwuk | *ground fern in burnt area* | vegetative matter | 1 | 30 | 0.0060 |
| semak pi | Semak | *fern* | pith | 1 | 5 | 0.0010 |
| semut in | Semut | *Lazius* sp. | insects | 2 | 564 | 0.1129 |
| soil ot | Soil | NA | other | 1 | 23 | 0.0046 |
| suli fl | Suli | *Etlingera triorgyalis* | flowers | NA | 3 | 0.0006 |
| suli fr | Suli | *Etlingera triorgyalis* | fruits | 1 | 31 | 0.0062 |
| suli ve | Suli | *Etlingera triorgyalis* | vegetative matter | 4 | 719 | 0.1439 |
| tagula bk | Tagula | *Alseodaphne* sp. | bark | 3 | 3 | 0.0006 |
| tagula daun kecil fr | Tagula Daun Kecil | *Litsea* sp. | fruits | 1 | 18 | 0.0036 |
| tagula fr | Tagula | *Alseodaphne* sp. | fruits | 1 | 1076 | 0.2153 |
| tagula lv | Tagula | *Alseodaphne* sp. | leaves | 0 | 6 | 0.0012 |
| takapal lv | Takapal |  | leaves | 0 | 14 | 0.0028 |
| takapal ve | Takapal |  | vegetative matter | NA | 2 | 0.0004 |
| takurak fr | Takurak | *Castanopsis cf. foxworthyii/jaherii* | fruits | NA | 12 | 0.0024 |
| tampa rajang lv | Tampa Rajang |  | leaves | 0 | 2 | 0.0004 |
| tampahening fr | Tampahening |  | fruits | NA | 4 | 0.0008 |
| tampang fl | Tampang | *Artocarpus dadak* | flowers | NA | 2 | 0.0004 |
| tampang fr | Tampang | *Artocarpus dadak* | fruits | 1 | 1658 | 0.3318 |
| tampang lv | Tampang | *Artocarpus dadak* | leaves | 0 | 3 | 0.0006 |
| tantimun fl | Tantimun | *Tetrameristra glabra* | flowers | 0 | 28 | 0.0056 |
| tantimun fr | Tantimun | *Tetrameristra glabra* | fruits | 2 | 7732 | 1.5473 |
| tantimun lv | Tantimun | *Tetrameristra glabra* | leaves | 0 | 2 | 0.0004 |
| tapuhut batu fr | Tapuhut Batu | *Syzigium* sp. | fruits | NA | 19 | 0.0038 |
| tapuhut merah fr | Tapuhut Merah | *Syzigium* sp. | fruits | 0 | 1 | 0.0002 |
| tapuhut putih fl | Tapuhut Putih |  | flowers | NA | 4 | 0.0008 |
| tapuhut putih fr | Tapuhut Putih |  | fruits | 1 | 5001 | 1.0008 |
| tapuhut putih lv | Tapuhut Putih |  | leaves | 0 | 24 | 0.0048 |
| tarantang bk | Tarantang | *Campnosperma coriaceum* | bark | 3 | 2 | 0.0004 |
| tarantang fr | Tarantang | *Campnosperma coriaceum* | fruits | 0 | 4315 | 0.8635 |
| tarantang lv | Tarantang | *Campnosperma coriaceum* | leaves | 0 | 1 | 0.0002 |
| tatumbu kalepang fr | Tatumbu Kalepang | *Syzigium* sp. | fruits | NA | 6 | 0.0012 |
| tatumbu kasar fl | Tatumbu Kasar | *Syzigium cf. Garcinifolia* | flowers | 0 | 89 | 0.0178 |
| tatumbu kasar fr | Tatumbu Kasar | *Syzigium cf. Garcinifolia* | fruits | 2 | 2588 | 0.5179 |
| tatumbu pohon merah fl | Tatumbu Pohon Merah | *Syzigium* sp. | flowers | NA | 1 | 0.0002 |
| tatumbu pohon merah fr | Tatumbu Pohon Merah | *Syzigium* sp. | fruits | 1 | 961 | 0.1923 |
| tatumbu pohon merah lv | Tatumbu Pohon Merah | *Syzigium* sp. | leaves | 0 | 3 | 0.0006 |
| tatumbu putih fl | Tatumbu Putih | *Syzigium* sp. | flowers | NA | 7 | 0.0014 |
| tatumbu putih fr | Tatumbu Putih | *Syzigium* sp. | fruits | 1 | 1599 | 0.3200 |
| tatumbu putih lv | Tatumbu Putih | *Syzigium* sp. | leaves | 0 | 4 | 0.0008 |
| tawon in | Tawon |  | insects | 3 | 1 | 0.0002 |
| telur burung ot | Telur burung (Bird egg) |  | other | 3 | 1 | 0.0002 |
| tilap fr | Tilap | *Artocarpus elasticus* | fruits | 1 | 172 | 0.0344 |
| tilap lv | Tilap | *Artocarpus elasticus* | leaves | 0 | 22 | 0.0044 |
| tumih lv | Tumih | *Combretocarpus rotundatus* | leaves | 0 | 34 | 0.0068 |
| tutup kabali fl | Tutup Kabali | *Diospyros pseudo-malabarica* | flowers | 0 | 5 | 0.0010 |
| tutup kabali fr | Tutup Kabali | *Diospyros pseudo-malabarica* | fruits | 3 | 36383 | 7.2807 |
| tutup kabali lv | Tutup Kabali | *Diospyros pseudo-malabarica* | leaves | 0 | 675 | 0.1351 |
| ulat in | Ulat |  | insects | 2 | 2603 | 0.5209 |

**Table ESM 2.** **Processing steps of food items.** The most frequent combinations of the different processing steps and descriptions of the corresponding food items with local and scientific names of example species at Tuanan (after Schuppli et al. 2016).

| **Nr** | **Processing steps** | **Food item types** | **Examples** |
| --- | --- | --- | --- |
| **0** | Pick | Fruits and flowers where everything is eaten | Lunuk (*Ficus* sp.), Tapuhut Putih (*Syzigium* sp.), Nyatoh Puntik (*Palaquium pseudorostrum*) |
| **1** | a) Pick, bite off | a) Fruits and flowers where a small outer part is bitten off after picking, discarded, and the remaining parts are eaten | a) Nyatoh undus buah merah (*Palaquium ridleyi*), Katiau (*Madhuca motleyana*), Mangkinang Blawau (*Elaeocarpus* sp.) |
|  | b) Pick, drop | b) Fruits and flowers where only the sap is ingested and all other parts are discarded after chewing | b) Rewui (*Microcos* sp.), Piais (*Nephelium mangayi*), Tampang (*Artocarpus dadak*) |
| **2** | a) Pick, peel, spit out | a) Fruits where the pulp is eaten while the skin and the seed are discarded | a) Papung (*Sandoricum borneense*) |
|  | b) Pick, bite in half, scrape flesh out | b) Hard-shell fruits where pulp and seeds are eaten but the empty pod is discarded | b) Lewang (*Pouteria cf. malaccensis*) |
|  | c) Pick, turn repeatedly in mouth, drop seed and skin layers | c) Fruits with edible flesh tightly attached to an inedible seed and thin skin | c) Enyak Beruk (*Syzygium* sp.), Nyatoh undus buah besar (*Palaquium cochlearifolium*), Tantimun unripe (*Tetrameristra glabra*) |
|  | d) Pick, pop pod open, extract seed | d) Fruit pods with an edible seed enclosed | d) Akar Kamunda (*Leucomphalos callicarpus*) |
| **3** | a) Pick, bite in half, scrape flesh out, spit out | a) Hard shell fruits where the pulp and seeds are eaten but the empty pod and seeds are discarded | a) Karandau Putih (*Blumeodendron kurzii*), Tutup Kabali (Diospyros pseudo-malabarica) |
|  | b) Collect substrate, scratch or bite open, suck | b) Insects embedded in wood or other substrate | b) Ants (*Formica* sp.), Termites (*Termitidae* sp.) |
|  | c) Bite piece of bark loose, rip or strip it off, scrape inner bark off | c) Inner bark (i.e., cambium/phloem) | c) Maruang (*Myristica lowiana*), Pantung (*Dyera lowii*) |
| **4** | 1. Pick, bite tip off, pull string off of pod to open it, turn pod open, extract seed | a) Bean-like fruits with inedible skin but edible seeds | a) Pantung (*Dyera lowii*) |

**Table ESM 3 Distribution of the different processing steps.** The percentage the different processing steps form over all 318 of food items that were identified in the study and the percentage of time the adult focal individuals spent feeding on items of the different processing steps (assessed via a total of 499,720 feeding bouts)

| **Processing step** | **Percent of all food items** | **Percent feeding bouts** |
| --- | --- | --- |
| 0 | 38.65 | 30.40 |
| 1 | 9.20 | 11.31 |
| 2 | 9.51 | 34.47 |
| 3 | 12.58 | 19.77 |
| 4 | 0.92 | 2.59 |
| NA | 29.14 | 1.46 |

**Table ESM 4 Sex differences in foraging time allocation of adult females and flanged males:** The effects of sex on the length of the daily active period, analyzed with a GLMM with Gaussian family distribution. R^2^ refers to conditional delta R^2^. Significant P – values of predictors are indicated with bold font

| **Response Variable** | **Factor** | **Factor type** | **Estimate** | **Std-Error** | **P-value** | **R^2^** |
| --- | --- | --- | --- | --- | --- | --- |
| Daily active hours | Intercept | Intercept | 11.235 | 0.096 | <0.001 | 0.352 |
|  | Sex (m) | Predictor | -0.346 | 0.136 | **0.011** |  |
|  | Year month | Random | - | - | - |  |
|  | Individual | Random | - | - | - |  |

**Table ESM 5** **Details on the Michaelis-Menten fits:** Parameters of the Michaelis-Menten fits used for (a) the overall analysis of sex differences in overall diet repertoires (Fig. 2, table 2) and the analyses used for estimating the total diet repertoire sizes of the adult females and flanged males using (b) the full data set including all follow hours of each individual (Fig. 3a) and (c) using the reduced data set including the minimal follow hours (i.e., 625 hours on each individual, Fig. 3 b). In our application of the Michaelis-Menten equation, parameter Vm is representative to the estimated total repertoire size and parameter K of the estimated number of follow hours at which half of the total repertoire will have been recorded

| **Nr.** | **Response Variable** | **Factor** | **Estimate** | **Std-Error** | **P-value** | **Residual standard error** |
| --- | --- | --- | --- | --- | --- | --- |
| a) | Food item count (all adult individuals) | K | 994.63 | 19.36 | <0.001 | 12.95 |
|  |  | Vm | 239.24 | 1.80 | <0.001 |  |
| b) | Food item count adult female Jinak | K | 180.10 | 14.77 | <0.001 | 4.11 |
|  |  | Vm | 118.01 | 3.71 | <0.001 |  |
|  | Food item count adult female Juni | K | 274.21 | 12.58 | <0.001 | 2.18 |
|  |  | Vm | 145.45 | 3.13 | <0.001 |  |
|  | Food item count adult female Kerry | K | 510.15 | 33.93 | <0.001 | 3.16 |
|  |  | Vm | 198.38 | 7.65 | <0.001 |  |
|  | Food item count adult female Kondor | K | 330.53 | 20.32 | <0.001 | 2.93 |
|  |  | Vm | 147.31 | 4.53 | <0.001 |  |
|  | Food item count adult female Mindy | K | 298.66 | 37.24 | <0.001 | 5.28 |
|  |  | Vm | 130.60 | 7.15 | <0.001 |  |
|  | Food item count adult female Milo | K | 273.87 | 24.39 | <0.001 | 3.93 |
|  |  | Vm | 115.05 | 4.37 | <0.001 |  |
|  | Food item count flanged male Helium | K | 249.69 | 37.69 | <0.001 | 5.35 |
|  |  | Vm | 108.73 | 8.43 | <0.001 |  |
|  | Food item count flanged male Henk | K | 219.80 | 9.83 | <0.001 | 2.38 |
|  |  | Vm | 121.16 | 2.29 | 0.001 |  |
|  | Food item count flanged male Niko | K | 265.62 | 8.54 | <0.001 | 1.92 |
|  |  | Vm | 129.88 | 1.97 | <0.001 |  |
|  | Food item count flanged male Otto | K | 517.35 | 53.93 | <0.001 | 3.16 |
|  |  | Vm | 130.79 | 8.09 | <0.001 |  |
|  | Food item count flanged male Sugus | K | 181.27 | 22.63 | <0.001 | 2.41 |
|  |  | Vm | 86.65 | 5.60 | <0.001 |  |
|  | Food item count flanged male Tomi | K | 361.79 | 31.79 | <0.001 | 2.81 |
|  |  | Vm | 102.33 | 4.34 | <0.001 |  |
|  | Food item count flanged male Wodan | K | 128.07 | 8.14 | <0.001 | 2.91 |
|  |  | Vm | 103.39 | 2.56 | <0.001 |  |
| c) | Food item count adult female Jinak | K | 879.50 | 39.47 | <0.001 | 10.01 |
|  |  | Vm | 228.52 | 3.13 | <0.001 |  |
|  | Food item count adult female Juni | K | 997.28 | 36.45 | <0.001 | 9.56 |
|  |  | Vm | 259.04 | 3.11 | <0.001 |  |
|  | Food item count adult female Kerry | K | 679.47 | 24.78 | <0.001 | 8.90 |
|  |  | Vm | 217.50 | 2.25 | <0.001 |  |
|  | Food item count adult female Kondor | K | 301.51 | 10.56 | <0.001 | 2.72 |
|  |  | Vm | 139.95 | 1.92 | <0.001 |  |
|  | Food item count adult female Mindy | K | 949.92 | 19.92 | <0.001 | 5.56 |
|  |  | Vm | 239.71 | 1.62 | <0.001 |  |
|  | Food item count adult female Milo | K | 848.02 | 43.74 | <0.001 | 5.66 |
|  |  | Vm | 209.52 | 4.89 | <0.001 |  |
|  | Food item count flanged male Helium | K | 249.69 | 37.69 | <0.001 | 5.35 |
|  |  | Vm | 108.73 | 8.43 | <0.001 |  |
|  | Food item count flanged male Henk | K | 232.70 | 10.53 | <0.001 | 2.62 |
|  |  | Vm | 124.85 | 2.33 | <0.001 |  |
|  | Food item count flanged male Niko | K | 309.10 | 6.73 | <0.001 | 2.35 |
|  |  | Vm | 140.27 | 1.13 | <0.001 |  |
|  | Food item count flanged male Otto | K | 499.20 | 34.00 | <0.001 | 3.04 |
|  |  | Vm | 127.71 | 5.62 | <0.001 |  |
|  | Food item count flanged male Tomi | K | 860.53 | 62.40 | <0.001 | 4.11 |
|  |  | Vm | 170.24 | 6.75 | <0.001 |  |
|  | Food item count flanged male Sugus | K | 181.27 | 22.63 | <0.001 | 2.41 |
|  |  | Vm | 86.65 | 5.60 | <0.001 |  |
|  | Food item count flanged male Wodan | K | 198.82 | 12.82 | <0.001 | 6.67 |
|  |  | Vm | 123.43 | 2.11 | <0.001 |  |
